# High fidelity CRISPR/Cas9 increases precise monoallelic and biallelic editing events in primordial germ cells

**DOI:** 10.1038/s41598-018-33244-x

**Published:** 2018-10-11

**Authors:** Alewo Idoko-Akoh, Lorna Taylor, Helen M. Sang, Michael J. McGrew

**Affiliations:** The Roslin Institute and Royal (Dick) School of Veterinary Studies, University of Edinburgh, Easter Bush Campus, Midlothian, EH25 9RG UK

## Abstract

Primordial germ cells (PGCs), the embryonic precursors of the sperm and egg, are used for the introduction of genetic modifications into avian genome. Introduction of small defined sequences using genome editing has not been demonstrated in bird species. Here, we compared oligonucleotide-mediated HDR using wild type SpCas9 (SpCas9-WT) and high fidelity SpCas9-HF1 in PGCs and show that many loci in chicken PGCs can be precise edited using donors containing CRISPR/Cas9-blocking mutations positioned in the protospacer adjacent motif (PAM). However, targeting was more efficient using SpCas9-HF1 when mutations were introduced only into the gRNA target sequence. We subsequently employed an eGFP-to-BFP conversion assay, to directly compare HDR mediated by SpCas9-WT and SpCas9-HF1 and discovered that SpCas9-HF1 increases HDR while reducing INDEL formation. Furthermore, SpCas9-HF1 increases the frequency of single allele editing in comparison to SpCas9-WT. We used SpCas9-HF1 to demonstrate the introduction of monoallelic and biallelic point mutations into the *FGF20* gene and generate clonal populations of edited PGCs with defined homozygous and heterozygous genotypes. Our results demonstrate the use of oligonucleotide donors and high fidelity CRISPR/Cas9 variants to perform precise genome editing with high efficiency in PGCs.

## Introduction

The chicken is a useful animal model for biological research and can be used to produce biopharmaceutical products that cannot be produced in mammalian bioreactor systems^1,2^. Chicken meat and eggs derived from 70 billion chickens yearly are an important source of high quality protein, vitamins and minerals in the global economy^3^. The ability to precisely edit the chicken genome to introduce or test genetic variants will aid the study of gene function, define combinatorial allelic contribution to disease resistance/resilience and production phenotypes and will lead to the uncovering of beneficial alleles which could be introduced into breeding programmes for the improvement of poultry welfare and sustainable production.

The application of precision genome editing to bird species has failed to keep pace with that of other mammalian species. In mammals, germline genetic engineering may be achieved through pronuclear injection of the zygote^4^, injection of genetically modified embryonic stem cells into the blastocyst^5^, and somatic cell nuclear transfer of genetically modified somatic cells (SCNT)^6,7^. These methods are not regularly practised in bird species because the single-cell zygote is difficult to access and manipulate and cultured avian embryonic stem cells do not contribute to the formation of the germ lineage^8,9^. In contrast to other species, heritable genetic changes may be introduced into chicken through the genetic manipulation of primordial germ cells (PGCs), the stem cell precursors of the sperm and egg, which can be propagated in culture and will contribute to the germline when reintroduced into surrogate host chick embryos^10–12^.

Targeted genetic modification in chicken PGCs was first demonstrated through classical gene targeting by homologous recombination (HR) to generate immunoglobulin-knockout chickens^13^. The observation that DNA Double-stranded breaks (DSBs) stimulate and increase the frequency of HR led to the development of artificial site-specific nucleases including ZFNs, TALENs and CRISPR/Cas9 with the goal of improving the efficiency of site-specific gene targeting^14,15^. Artificial site-specific nucleases are guided to a specific genomic site by programmable DNA-binding modules where they create a DSB. The cleaved DNA is immediately repaired by either non-homologous end-joining (NHEJ) or the HR pathway^16–18^. NHEJ is the predominant pathway that repairs DSBs that occur in all phases of the cell cycle and often leads to the generation of insertion/deletion (INDEL) mutations^19^. The HR or homology-directed repair (HDR) pathway is active in the S and G2 phase of the cell cycle and is used to repair a double-stranded DNA break when there is an available DNA donor containing a region that is homologous to the region surrounding the severed DNA ends^16,20,21^. The high fidelity of HDR is constrained by the nucleotide composition of the repair template and this constraint is exploited in genome engineering to introduce a desired nucleotide change. TALENs and CRISPR/Cas9-mediated NHEJ have been used to produce several knockout-chickens through the generation of INDELs in chicken PGCs^22,23^. Homologous recombination mediated by TALENs and CRISPR/Cas9 have also been performed in chicken PGCs to introduce targeted transgenes^24,25^. However, the use of site-specific nucleases to perform precision editing of a single to few nucleotides has not been demonstrated in avian species.

Genome editing mediated by CRISPR/Cas9 and short single stranded oligodeoxynucleotides (ssODN) donors has been used to perform small precise genetic changes in many cell types and organisms^26–37^ and in a chicken somatic cell line^38^. However, use of ssODN donors for gene correction can be toxic to cells by causing a G2/M cell cycle arrest^39^, and activating cellular immune responses^40^. HDR targeting therefore requires careful optimization for each cell type. Following CRISPR/Cas9-induced DSBs, DNA repair with ssODN donors occurs through the synthesis-dependent strand annealing (SDSA) pathway of HDR^41^. However, the accuracy of HDR editing may be distorted by the incorporation of INDELs at the target site in a second round of repair due to re-cleaving by CRISPR/Cas9^42,43^. Previous studies have shown that the introduction of Cas9-blocking mutations in the PAM are effective in preventing re-editing of genetic loci while blocking mutations positioned in the gRNA target sequence have variable efficacy^35^.

High fidelity CRISPR/Cas9 nucleases with improved specificity have been developed to reduce the frequency of off-target events associated with wild type *Streptococcus pyogenes* Cas9 (SpCas9-WT)^44–46^. These high fidelity Cas9 variants harbour amino acid substitutions that significantly reduce activation of cleavage at target sites that are not perfectly complementary to the gRNA sequence. Here, we investigated a high fidelity Cas9 variant, SpCas9-HF1^45^, for introducing defined nucleotide changes in chicken PGCs using ssODN donors. First, we optimised the use of ssODN donors as repair templates for CRISPR/Cas9-mediated HDR in cultured chicken PGCs. We then directly compared HDR editing between SpCas9-WT and SpCas9-HF1 using ssODN donors containing CRISPR/Cas9-blocking mutations positioned in the PAM and show that many loci in chicken PGCs can be efficiently edited using SpCas9-HF1. Using ssODN donors containing mutations in the guide sequence only, we also showed that SpCas9-HF1 is more efficient than SpCas9-WT in introducing precise genome edits in the absence of CRISPR/Cas9-blocking mutation in the PAM. We subsequently used a eGFP-to-BFP conversion assay^47^ to directly compare HDR mediated by SpCas9-WT and SpCas9-HF1 and found that SpCas9-HF1 increases the efficiency of accurate HDR editing while reducing INDEL formation at the target site. Finally, we combined SpCas9-HF1 and ssODN donors to demonstrate precise biallelic and monoallelic introduction of the chicken *scaleless* genetic variant associated with the heat tolerance featherless phenotype by introducing two defined nucleotide substitutions into the *FGF20* gene^48–50^. Our results demonstrate the use of high fidelity CRISPR/Cas9 variants to perform precise HDR genome editing with high efficiency in chicken PGCs.

## Results

### High fidelity Cas9 variant, SpCas9-HF1, shows efficient HDR editing in chicken PGCs

**Figure 1:**
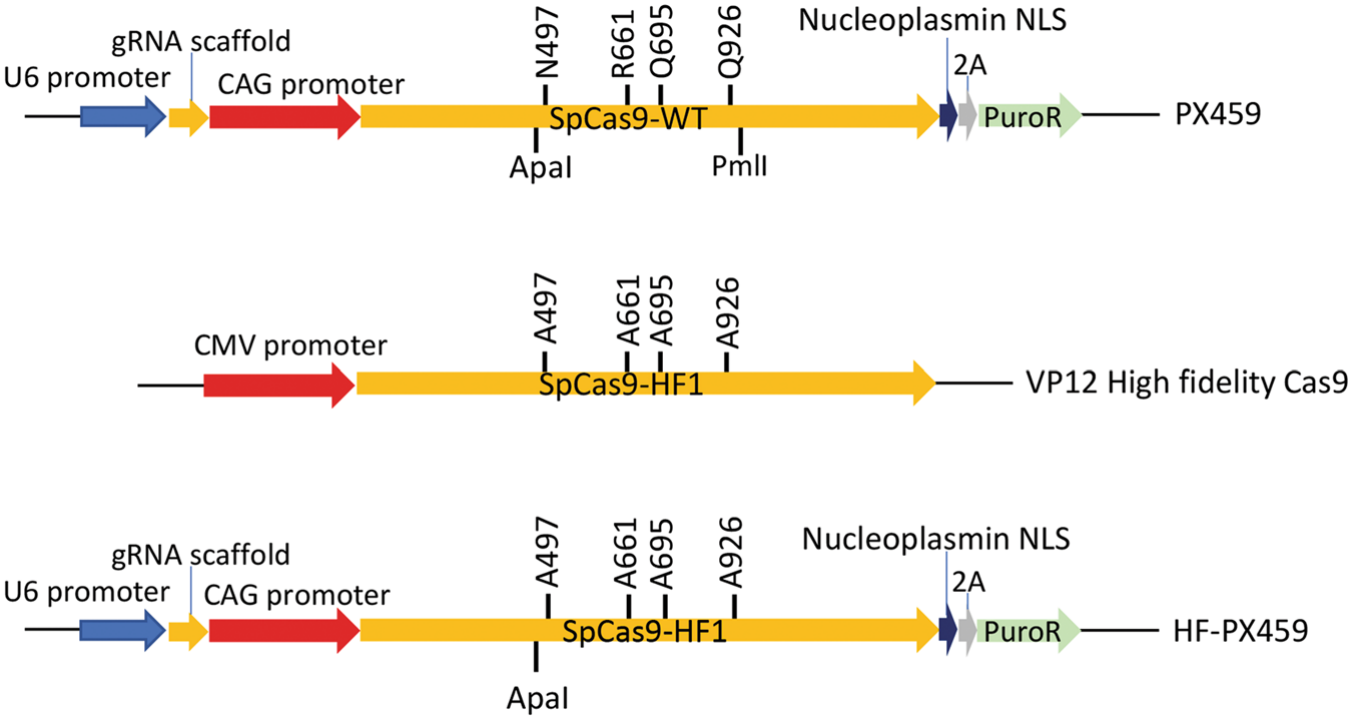
Schematic representation of CRISPR/Cas9 vectors used in this study. HF-PX459 and PX459 vectors differ by only four amino acids in the encoded Cas9 protein. HF-PX459 vector encodes SpCas9-HF1 while PX459 encodes SpCas9-WT. The U6 promoter drives sgRNA expression while expression of Cas9 protein and puromycin resistance (PuroR) is driven by the CAG promoter.

We first tested SpCas9-WT and high fidelity SpCas9-HF1 to edit multiple loci in chicken PGCs. SpCas9-HF1 contains 4 amino acid substitutions that prevent activation of the nuclease at mismatched targets^45^. To directly compare SpCas9-HF1 and SpCas9-WT, we transferred the codon-changing mutations from the VP12 vector which encodes SpCas9-HF1 into PX459 vector which encodes a mammalian-codon optimised SpCas9-WT as well as puromycin resistance^33^ (Fig. 1). We named this modified vector HF-PX459.

**Figure 2:**
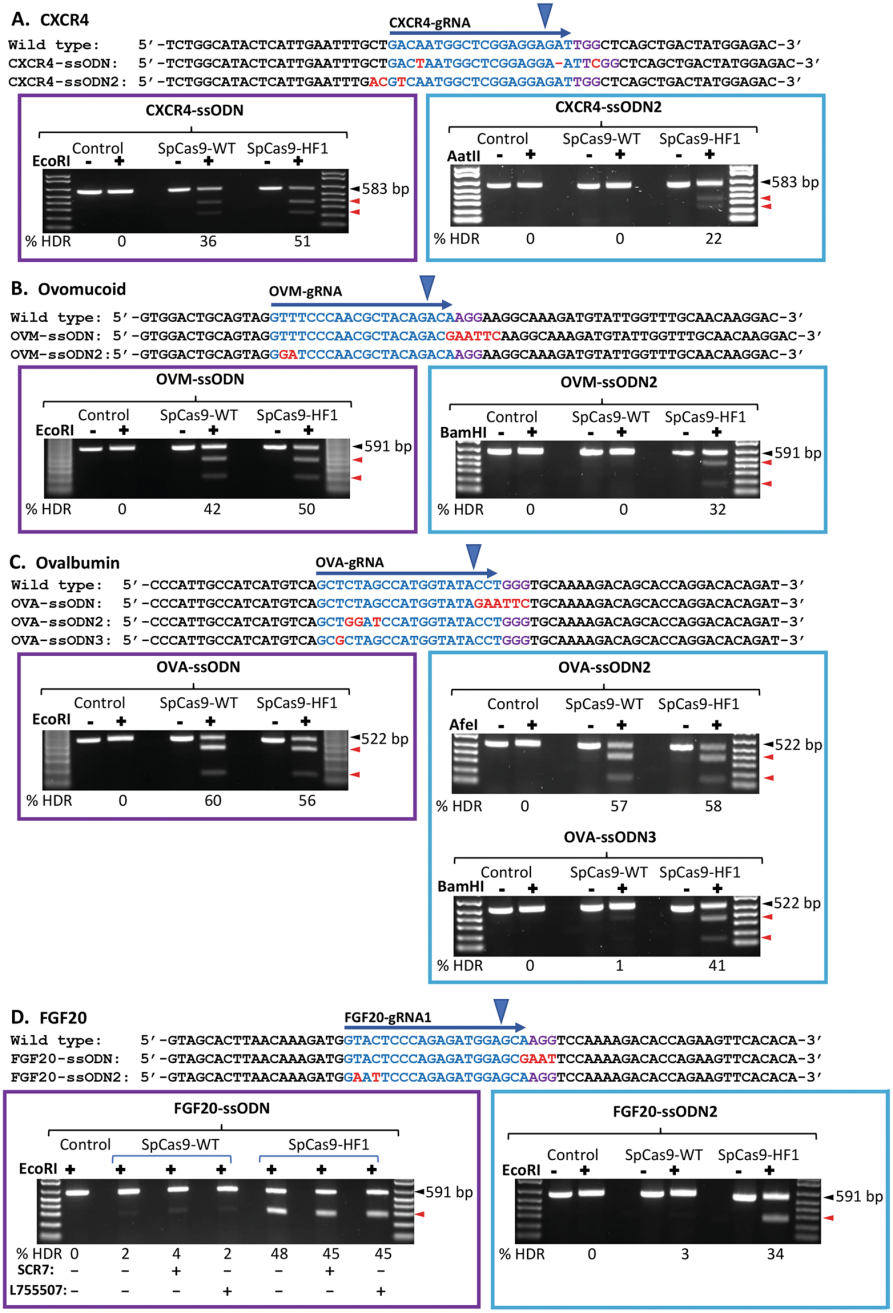
Efficient oligonucleotide-template HDR editing across multiple loci using SpCas9-HF1. Left panels: PAM-region mutated templates. Right panels: Guide-region mutated templates. All HDR ssODN donors were complementary to the gRNA target strand and were symmetric around the cut site except for FGF20-ssODN. gRNA sequences are highlighted in blue. PAMs are highlighted in purple. Blue arrowheads indicate Cas9 cleavage sites. Insertions, deletions and substitutions in HDR donors are highlighted in red. Black arrowheads indicate undigested PCR substrate. Red arrowheads indicate digested PCR products. (**−**) untreated substrate. (+) treated substrate. (See Figure S9 for uncropped images).

To compare HDR editing between SpCas9-WT and SpCas9-HF1 in chicken PGCs, we designed gRNAs to target exon 3 of *FGF20* (*FGF20-gRNA1*) and exon 2 of *CXCR4* (*CXCR4-gRNA*), and used previously described gRNAs to target exon 3 of ovalbumin (*OVA*) and exon 1 of ovomucoid (*OVM*) (*OVA-gRNA* for *ovalbumin* and *OVM-gRNA* for *ovomucoid*)^23^ (Fig. 2). In order to directly analyse HDR efficiency without sequencing PCR products, we used antisense repair templates that introduce an *Eco*RI recognition site for RFLP analysis^51,52^. *CXCR4* is expressed in chicken PGCs while *FGF20*, *ovalbumin* and *ovomucoid* are transcriptionally inactive. To target each locus, we used ssODN donors containing mutations of the gRNA seed sequence and PAM to insert an *Eco*RI recognition sequence (122-nt *CXCR4-ssODN* for *CXCR4*, 126-nt *FGF20-ssODN* for *FGF20*, 127-nt *OVA-ssODN* for *ovalbumin* and 128-nt *OVM-ssODN* for *ovomucoid*; Fig. 2). We co-transfected the corresponding ssODN donor and gRNA with SpCas9-HF1 or SpCas9-WT into PGCs and then treated with puromycin to select for Cas9-transfected cells. We performed two independent targeting experiments for each locus. To analyse HDR, we PCR amplified the target site and performed *Eco*RI RFLP digest assay on the PCR products to estimate HDR efficiency. In *CXCR4*, we observed an average HDR efficiency of 50.5% in PGCs targeted with SpCas9-HF1 and 35.5% with SpCas9-WT (Fig. 2A and Supplementary Fig. S2). For *ovomucoid*, the average HDR efficiency was 42.5% using SpCas9-HF1 and 39.5% with SpCas9-WT (Fig. 2B and Supplementary Fig. S2), whereas the average HDR efficiency was 63.5% with SpCas9-HF1 and 62.5% with SpCas9-WT for *ovalbumin* (Fig. 2C and Supplementary Fig. S2). For *FGF20*, we also tested the NHEJ inhibitors, SCR7 and L755507, reported to increase HDR efficiency^34,53^. We observed an average HDR efficiency of 48.5% and 3.5% with SpCas9-HF1 and SpCas9-WT respectively without using NHEJ inhibitors while no HDR improvement was observed with either SCR7 or L755507 treatment (Fig. 2D and Supplementary Fig. S2). Thus, NHEJ inhibitors do not increase targeting efficiency in PGCs and targeting efficiencies were equal or slightly better using SpCas9-HF1 in combination with donor containing PAM mutations.

We next tested whether introducing single-nucleotide blocking mutations into the PAM-distal region of the gRNA target sequence was sufficient to achieve high HDR without introducing a blocking mutation in the PAM. Cleavage activity of Cas9 was shown to be severely reduced when mismatches are present in the seed region (first 8–12 nucleotides proximal of the PAM) of the gRNA target sequence^54–57^. However, mismatches were tolerated in the PAM-distal non-seed region of the gRNA target and are associated with off-target mutagenesis by SpCas9-WT^58–60^. To test the efficiency of accurate HDR when introducing single nucleotide mutations into the non-seed region of the gRNA target sequence, we designed ssODN donors containing one to three substitutions of the last 14–20 PAM-distal nucleotides (120-nt *CXCR4-ssODN2* for *CXCR4*, 128-nt *OVM-ssODN2* for *ovomucoid*, 127-nt *OVA-ssODN2* for *ovalbumin*, 127-nt *OVA-ssODN3* for *ovalbumin* and 126-nt *FGF20-ssODN2 for FGF20*; Fig. 2, right panels). The substitutions introduce a restriction site for RFLP analysis. We transfected and analysed cells as above. For *CXCR4*, we observed an average HDR efficiency of 17.5% using SpCas9-HF1 and 0.0% with SpCas9-WT when *CXCR4-ssODN2* was supplied as repair template (Fig. 2A and Supplementary Fig. S2). For *ovomucoid*, the average HDR efficiency was 34.0% using SpCas9-HF1 and 0.5% with SpCas9-WT when *OVM-ssODN2* was supplied as repair template (Fig. 2B and Supplementary Fig. S2). For *ovalbumin*, the average HDR efficiency was 58.5% using SpCas9-HF1 and 53.5% using SpCas9-WT when *OVA-ssODN2* was supplied as repair template (Fig. 2C and Supplementary Fig. S2). When *OVA-ssODN3* was used as a repair template containing a single base pair change in the PAM distal guide region, we observed an average HDR efficiency of 43.5% using SpCas9-HF1 and 1.0% using SpCas9-WT (Fig. 2C and Supplementary Fig. S2). For *FGF20*, we observed an average HDR efficiency of 37.5% with SpCas9-HF1 and 3.5% with SpCas9-WT when *FGF20-ssODN2* was supplied as repair template (Fig. 2D and Supplementary Fig. S2). We also compared symmetrical and asymmetrical ssODNs as well as a double stranded repair template carried in plasmid but observed similar levels of HDR at *FGF20* (data not shown). Our results show that SpCas9-HF1, in comparison to SpCas9-WT, is effective for achieving precise introduction of single nucleotide changes into the non-seed region of the gRNA target sequence. These results demonstrate the accuracy and versatility of SpCas9-HF1.

### SpCas9-HF1 reduces INDEL formation at target site in comparison to SpCas9-WT

**Figure 3:**
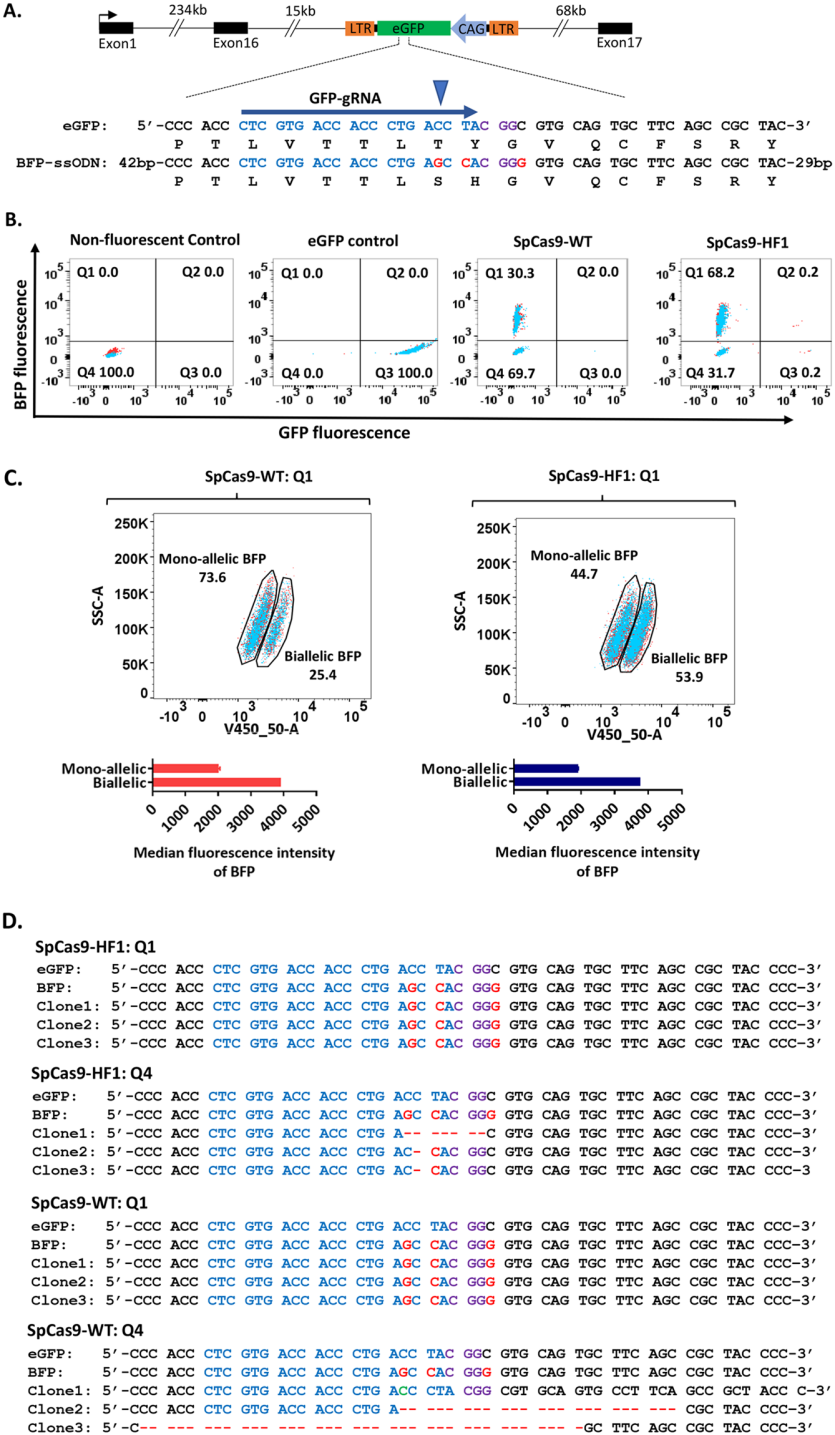
SpCas9-HF1 increases HDR by reducing INDEL formation. (**A**) An eGFP expression cassette was integrated in the MAD1L1 gene. CAG promoter drives eGFP expression. The gRNA sequence is highlighted in blue while the PAM is highlighted in purple. Blue arrowhead indicates Cas9 cleavage site. Substitutions in the BFP-ssODN donor are highlighted in red. BFP-ssODN is a 122-nt HDR template that is complementary to the target strand. (**B**) Flow cytometric detection of BFP-expressing PGCs and non-fluorescent PGCs to quantify HDR and INDEL levels respectively. (**C**) Flow cytometric quantification of BFP-expression PGCs (top panels) and mean fluorescent intensity of BFP-expressing PGCs (bottom panels). (**D**) Alignment of sequences of cloned PCR products from PGCs in quadrants Q1 and Q4.

To better quantitate HDR and INDEL formation mediated by SpCas9-WT and SpCas9-HF1, we used an assay that converts enhanced green fluorescent protein (*eGFP*) to blue fluorescent protein (*BFP*) after editing events^47^. The eGFP-to-BFP conversion assay simultaneously quantifies total HDR and NHEJ events in a targeted population. We targeted PGCs isolated from homozygous transgenic chicken ubiquitously and constitutively expressing *eGFP* (GFP-PGCs; Fig. 3A) with a validated gRNA (*GFP-gRNA*) which was co-delivered with SpCas9-WT or SpCas9-HF1 and a ssODN donor carrying three nucleotide substitutions (*BFP-ssODN*; Fig. 3A) designed to convert eGFP to BFP^47^. In this case the 20 nucleotide *GFP-gRNA* begins with a C nucleotide which reduces transcription from the U6 promoter^61^. *BFP-ssODN* donor contains a C-to-G substitution that converts Threonine (T) to Serine (S), a T-to-C substitution that converts Tyrosine (Y) to Histidine (H) and a synonymous T-to-G substitution. The C-to-G substitution (the 1^st^ nucleotide of the gRNA seed sequence) and the T-to-C substitution (1^st^ nucleotide of the PAM) serve as Cas9-blocking mutations to prevent re-editing and increase HDR accuracy of SpCas9-WT. *eGFP* is converted to *BFP* by a Y66H amino acid substitution. Error-free editing of the *eGFP* sequence will lead to the expression of BFP while the presence of INDELs, even after recombinational repair, will result in no *BFP* or *eGFP* expression due to a shift in the reading frame. Before transfection, 99.9% of gated living cells expressed *eGFP*. Transfection with SpCas9-WT vector resulted in 30.3% of PGCs expressing BFP and the remaining 69.7% did not express GFP indicating INDEL formation. In contrast, transfection with SpCas9-HF1 vector resulted in 68.2% of PGCs expressing *BFP* and the remaining 31.7% did not express eGFP (Fig. 3B). The HDR and INDEL levels obtained with SpCas9-WT in this assay are consistent with reports by other researchers using human cell lines^47,62^. Two distinct populations of BFP-expressing PGCs were observed for both SpCas9-WT and SpCas9-HF1 (Fig. 3C, top panel) and determined their median fluorescent intensity (MFI). We determined that the population with the lower MFI of approximately 2000 units was monoallelic for *BFP* and contained INDELs on the second GFP allele while the population with an MFI of approximately 4000 units was biallelic for *BFP* by TIDE analysis^63^ of the PCR sequencing traces of these populations (Fig. 3C, bottom panel, Supplementary Fig. S3). Interestingly, we noticed that the proportion of BFP PGCs transfected with SpCas9-HF1 that was biallelic for BFP was 53.9% while 44.7% was monoallelic for BFP. For PGCs transfected with SpCas9-WT, 25.4% of BFP PGCs was biallelic for BFP while 73.6% was monoallelic for BFP. This indicates that SpCas9-HF1 increases the efficiency of biallelic HDR by up to two-fold by reducing INDEL formation. Also, the absence of PGCs expressing only *eGFP* or co-expressing *eGFP* and *BFP* is indicative of the high mutagenic activity of CRISPR/Cas9 and is similar to previous observation^47,62^. Our results show that using SpCas9-HF1 with ssODN donors containing Cas9-blocking mutations positioned in the gRNA sequence increases HDR levels by more than 2-fold with a concomitant decrease in INDEL formation in comparison to SpCas9-WT.

### SpCas9-HF1 efficiently introduces heterozygous biallelic edits in comparison to SpCas9-WT

**Figure 4:**
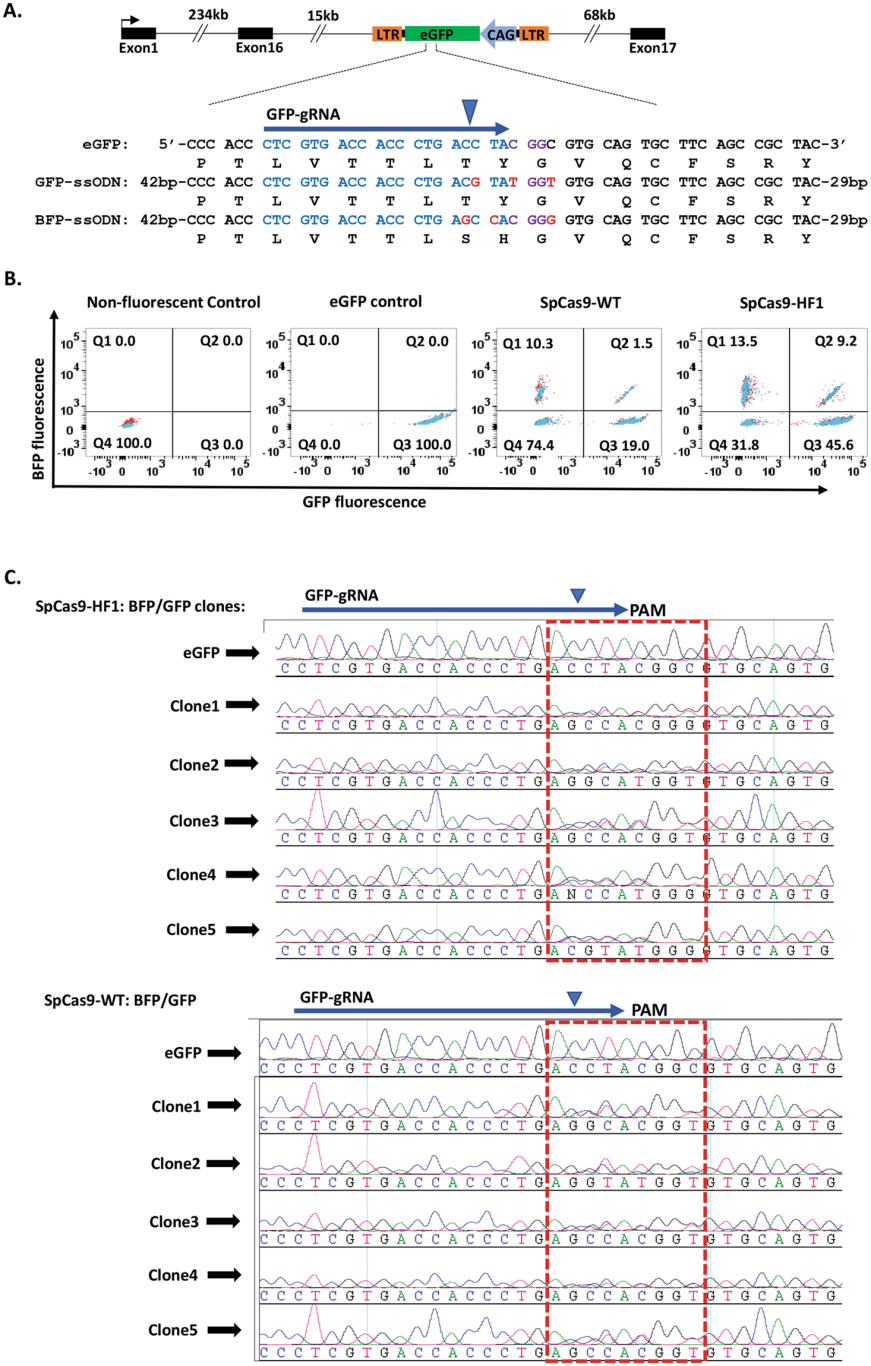
SpCas9-HF1 increases the frequency of single allele editing (**A**) GFP-ssODN contains three synonymous substitutions to preserve that amino acid sequence of eGFP. Substitutions in ssODN donors are highlighted in red. gRNA target sequence is coloured blue, PAM is highlighted in purple, blue arrowhead indicates Cas9 cleavage site. (**B**) Flow cytometric detection and quantification of PGCs co-expressing GFP and BFP. (**C**) Sanger sequencing traces of direct PCR products from representative isolated single cell clones co-expressing GFP and BFP. Red box encloses region containing edits.

In the experiment above (Fig. 3), we observed that less than 0.2% of PGCs were heterozygous for *eGFP* and *BFP* using SpCas9-HF1 while no BFP-eGFP heterozygotes were obtained using SpCas9-WT. This observation reflects the experimental difficulty in generating specific heterozygous mutations since most CRISPR/Cas9 editing events are biallelic and cells with monoallelic HDR edits usually contain INDELs on the second allele^35,64^. A strategy that has been employed for editing single alleles in human IPS cells uses a mixture of two ssODN donor templates containing Cas9-blocking mutations with an observed efficiency of 0.1%^35^. Since SpCas9-HF1 increases HDR levels as well as the efficiency of biallelic HDR by reducing INDEL formation (Fig. 3B and C), we reasoned that SpCas9-HF1 could increase the efficiency of editing individual alleles using two ssODN donors. We compared SpCas9-WT and SpCas9-HF1 by performing eGFP-to-BFP editing of single *eGFP* alleles in GFP-PGCs to produce eGFP/BFP heterozygote cells. We designed a second repair template (*GFP-ssODN*) containing three synonymous nucleotide substitutions to preserve the amino acid sequence of eGFP (Fig. 4A). GFP-PGCs were then transfected with SpCas9-WT or SpCas9-HF1 vectors and equimolar amounts of *GFP-ssODN* and *BFP-ssODN* donors and then analysed by flow cytometry for expression of *BFP* and *eGFP*. The results from two independent experiments are shown in Fig. 4B. 1.5% of PGCs targeted with SpCas9-WT co-expressed *eGFP* and *BFP* while 9.2% of PGCs targeted with SpCas9-HF1 were eGFP/BFP co-expressing cells reflecting an almost 7-fold increase in HDR frequency in comparison to SpCas9-WT. Direct sequencing of PCR products from single-cell clones co-expressing *eGFP* and *BFP* confirmed incorporation of nucleotide changes in ssODN donors into the individual *eGFP* alleles (Fig. 4C). Similar to our previous result (Fig. 3B), we observed that 74.4% of PGCs targeted with SpCas9-WT did not express *eGFP* or *BFP* in comparison to 31.8% for PGCs targeted with SpCas9-HF1 (Fig. 4B). These results illustrate that SpCas9-HF1 increases the frequency of editing individual alleles by increasing HDR efficiency while reducing INDEL formation.

### Precise biallelic introgression of a genetic variant into chicken PGCs

**Figure 5:**
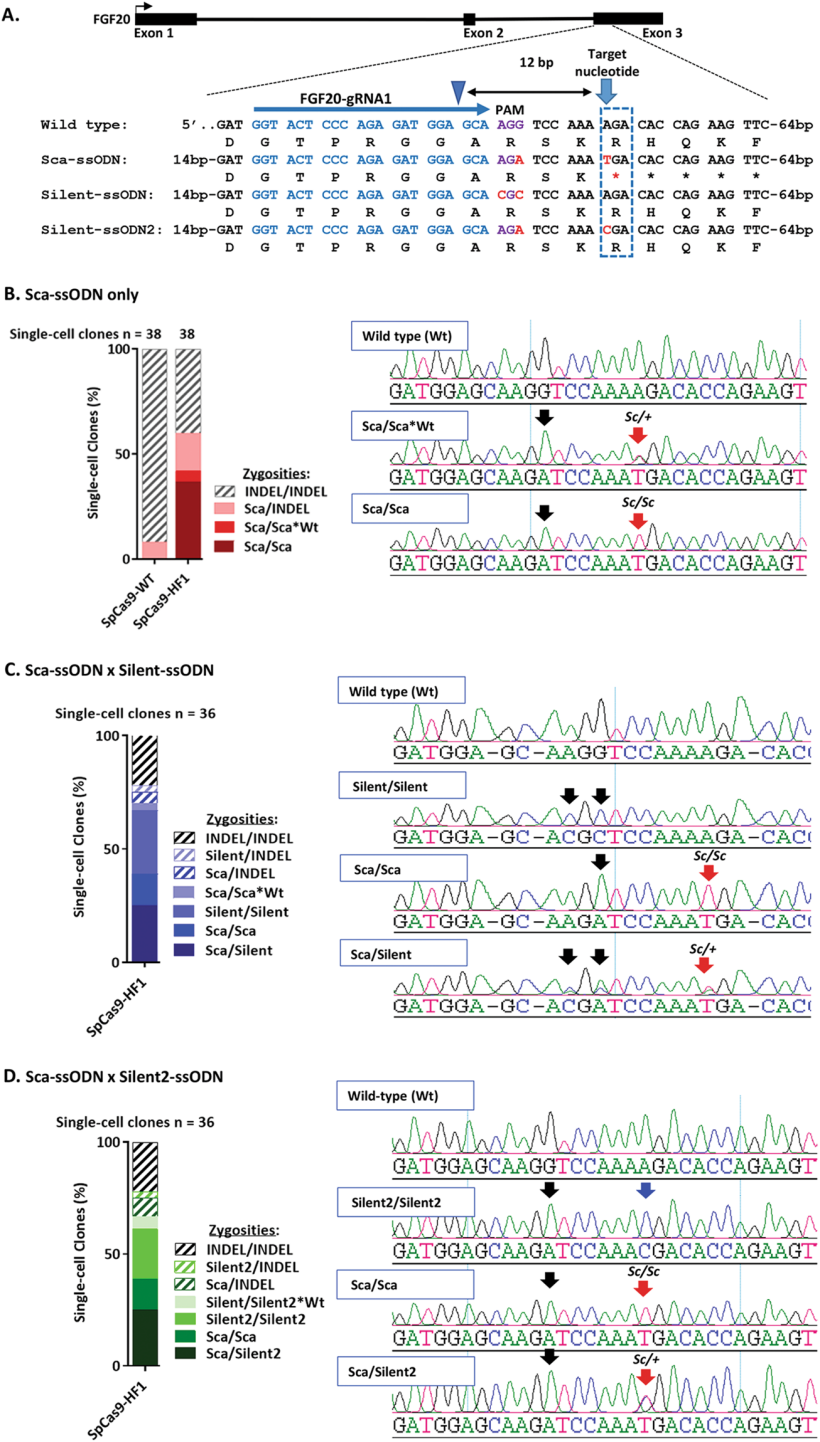
Introgression of *scaleless* 535*A* > T mutation into FGF20. (**A**) Strategy for introduction of 535*A* > T mutation into Exon 3. The gRNA directs cleavage 12 bp from the position of the targeted nucleotide change. Substitutions in ssODN donors are highlighted in red. PAM is highlighted in purple. Blue arrowhead indicates Cas9 cleavage site. All ssODN donors are complementary to the target strand. (**B**–**D**) Frequency of the mutation genotypes of isolated single-cell clones based on ssODN donor used for allelic repair. Representative sequence traces show mutation genotypes of HDR clones. Black arrows indicate PAM mutations, red arrows indicate *Sc* mutations, blue arrow shows biallelic 535*A* > C mutation.

We next demonstrated the introgression of specific genetic variants into PGCs. The *scaleless* mutation (*sc/sc*) is a single A-to-T substitution in exon 3 of *FGF20* (535A > T) which creates a premature stop codon resulting in a truncated FGF20 protein that leads to a complete loss of feather development^48^. We selected three gRNAs (Supplementary Fig. S1) targeting for the location of the *scaleless* variant but only gRNA1 (*FGF20-gRNA1*) containing a cut site that is 12 bp away from the target nucleotide was active with both SpCas9-WT and SpCas9-HF1 (Fig. 5A and Supplementary Fig. S1). We designed an ssODN donor (*Sca-ssODN*) containing a Cas9-blocking synonymous point mutation in the PAM (AGG → AGA) and the *scaleless* mutation (535A > T) which was 6 bp downstream of the 3′ end of the PAM (Fig. 5A). We anticipated that the 12 bp distance from the cut site to the edit site (cut-to-edit distance) would reduce editing accuracy. It has previously been shown that the efficiency of heterologous DNA incorporation is reduced as the distance between the site of edit and Cas9 cleavage site increases and the highest efficiency is achived within a distance of 8 to 10 bp^27,35,65^. To address the cut-to-edit distance, we used an asymmetric design for the ssODN donor containing a left homology arm (HA) of 36 bp and right HA of 91 bp to provide increased homology on the side containing the edited PAM and the 535A > *T* mutation. This asymmetric repair template design was previously described to increase HDR efficiency in human HEK293 and K562 cell lines by up to 60%^66^.

To introduce the 535A > *T* (*sc/sc*) gene variant, we transfected PGCs with *FGF20-gRNA1* and SpCas9-HF1 or SpCas9-WT with *Sca-ssODN* and then sequenced PCR products directly from 38 single-cell clonal populations isolated from two independent experiments to analyse their mutational status and zygosity. The results are shown in Table 1 and Fig. 5B.

**Table 1:** Edits of the FGF20 gene based on ssODN donor used for allelic repair in single-cell clones targeted with Sca-ssODN and SpCas9 or SpCas9-HF1 (Supplementary Figs S4 and S5).

Clone genotype	Sca/Sca	Sca/Sca*Wt	Sca/INDEL	INDEL/INDEL	Total
SpCas9-WT clones	0	0	3 (7.9%)	35 (92.1%)	38 (100%)
SpCas9-HF1 clones	14 (36.8%)	2 (5.3%)	7 (18.4%)	15 (39.5%)	38 (100%)
HDR	Biallelic	Biallelic	Monoallelic	N/A	N/A

We found that 7.9% of isolated clones transfected with SpCas9-WT contained precise monoallelic introduction of the PAM substitution and the *scaleless* mutation on one chromosome while the other chromosome contained INDELs. The other 92.1% of isolated clones contained INDELs on both chromosomes with no incorporation of the *scaleless* mutation. We note that this frequency is much higher than the INDEL frequency measured for this guide using the T7 endonuclease I assay (Fig. S1). We attribute this difference to inefficiencies of the T7 endonuclease I assay^67^. In contrast, 41.8% of single-cell clones transfected with SpCas9-HF1 were precise biallelic HDR clones. We discovered that 36.8% of the SpCas9-HF1 clones contained accurate biallelic incorporation of the PAM substitution and the 535A > *T* (*sc/sc*) mutation (Fig. 5B). We also noted that the PAM and 535A > *T* mutation was incorporated into only one chromosome (*sc/*+) in two SpCas9-HF1 clones (5.3% of isolated clones), while the second chromosome only contained the PAM substitution. Furthermore, we observed that 18.4% of the total SpCas9-HF1 clones were precise monoallelic HDR clones containing the PAM substitution and 535A > *T* mutation on one chromosome while the other chromosome contained INDELs. The high rate of biallelic editing is similar to observations in human IPSCs and mouse ES cells targeted with SpCas9-WT^35,64^. We noted that the 535A > *T* mutation was incorporated into 37 out of 39 HDR alleles (94.9%) in SpCas9-HF1 clones.

We next attempted to introduce the 535A > *T* gene variant into a single allele (*sc/*+) by providing two repair templates. Our previous result (Fig. 4B) showed that SpCas9-HF1 increases the overall efficiency of editing individual alleles using two ssODN donors whereas isolation of biallelically edited heterozygotes was barely detectable when SpCas9 was used. Consequently, we only tested SpCas9-HF1 in the following experiment. We designed two donors to introduce silent mutations on one allele while incorporating the 535*A* > *T* mutation into the second allele using Sca-ssODN (Fig. 5A). The first silent donor (Silent-ssODN) contained 2 synonymous substitutions in the PAM (AGG → CGC) to preserve the FGF20 amino acid sequence. In the second silent donor (Silent2-ssODN), the PAM mutation was the same as in *Sca-ssODN* (AGG → AGA) while a synonymous substitution 535*A* > *C* was made in the same position as the 535*A* > *T* mutation 12 bp from the cleavage site to maintain the cut-to-edit distance between templates. Since *Silent-ssODN* showed more complementarity to the target region than *Sca-ssODN* due to the 535*A* > *T* substitution, we asked whether the two silent repair templates would be used at different frequencies for allelic repair when used with *Sca-ssODN*.

We transfected SpCas9-HF1 with FGF20-gRNA1 and an equimolar mixture of *Sca-ssODN* donor and *Silent-ssODN* (Sca-ssODN/Silent-ssODN mixture) or an equimolar mixture of *Sca-ssODN* and *Silent2-ssODN* (Sca-ssODN/Silent2-ssODN mixture) for comparison. We performed two independent experiments and isolated a total of 18 single-cell clonal populations in each experiment. The results are shown in Tables 2 and 3 and Fig. 5C,D.

**Table 2:** Edits of the FGF20 gene based on ssODN donor used for allelic repair in single-cell clones targeted with SpCas9-HF1 and a mixture of Sca-ssODN and Silent-ssODN (Supplementary Fig. S6).

Clone genotype	Sca/Silent	Sca/Sca	Silent/Silent	Sca/Sca*Wt	Sca/INDEL	Silent/INDEL	INDEL/INDEL	Total
SpCas9-HF1 clones	9 (25.0%)	5 (13.9%)	10 (27.8%)	1 (2.8%)	2 (5.6%)	1 (2.8%)	8 (22.2%)	36 (100%)
HDR	Biallelic	Biallelic	Biallelic	Biallelic	Monoallelic	Monoallelic	N/A	N/A

**Table 3:** Edits of FGF20 gene based on ssODN donor used for allelic repair single-cell clones targeted with SpCas9-HF1 and a mixture of Sca-ssODN and Silent2-ssODN (Supplementary Fig. S7).

Clone genotype	Sca/Silent2	Sca/Sca	Silent2/Silent2	Silent2/Silent2*Wt	Sca/INDEL	Silent2/INDEL	INDEL/INDEL	Total
SpCas9-HF1 clones	9 (25.0%)	5 (13.9%)	8 (22.2%)	2 (5.6%)	3 (8.3%)	1 (2.8%)	8 (22.2%)	36 (100%)
HDR	Biallelic	Biallelic	Biallelic	Biallelic	Monoallelic	Monoallelic	N/A	N/A

Biallelic HDR editing with the co-incorporation of PAM mutations into the two alleles was observed in 69.5% of the clones targeted with the *Sca-ssODN*/*Silent-ssODN* mixture in contrast to 55.5% observed with the *Sca-ssODN*/*Silent2-ssODN* mixture (Fig. 5C). Remarkably, 25% of the clones contained the heterozygous edit for 535*A* > *T* (*sc/*+) in which the two alleles were independently repaired by the two ssODN templates (*Sca/Silent* and *Sca/Silent2;* Tables 2 and 3). 13.9% of the isolated clones targeted with the *Sca-ssODN*/*Silent-ssODN* mixture were biallelically repaired by *Sca-ssODN* (*Sca/Sca*) whereas 25.0% of the clones were biallelically repaired by *Silent-ssODN* (*Silent/Silent*) (Table 2 and Fig. 5C). One clone (Sca/Sca*Wt) was biallelically repaired by *Sca-ssODN* and was homozygous for the PAM substitution but heterozygous for 535*A* > *T* (*sc/*+) indicating partial introduction of edits in one chromosome (Table 2). We noted that 30 out of the 53 HDR alleles (56.6%) were repaired by Silent-ssODN when *Sca-ssODN* and *Silent-ssODN* were used together. Similarly, *Silent2-ssODN* repaired 57.7% of the 52 HDR alleles generated when it was used with *Sca-ssODN*. Using *Sca-ssODN* and *Silent2-ssODN* together, we also observed that 22.2% of the total isolated clones were biallelically repaired by *Silent2-ssODN* (*Silent2/Silent2*) while 13.9% were biallelically repaired by *Sca-ssODN* (*Sca/Sca* and Sca/Sca*Wt). We identified 2 clones biallelically harbouring the AGG → AGA PAM substitution on the two alleles but containing 535*A* > *C* mutation on only one allele indicating partial introduction of edits. While the proportion of biallelically edited heterozygous clones were the same using the two ssODN donor mixtures (Sca-ssODN/Silent-ssODN and Sca-ssODN/Silent2-ssODN mixtures), we observed that clones with monoallelic HDR contained INDELs on the other allele. The 535*A* > *T* substitution was incorporated into 22 out of 23 HDR alleles (95.7%) repaired by *Sca-ssODN* in clones targeted with *Sca-ssODN*/*Silent-ssODN* mixture. Similarly, the 535*A* > *T* substitution was incorporated into all 22 HDR alleles (100.0%) repaired by *Sca-ssODN* in clones targeted with *Sca-ssODN*/*Silent2-ssODN* mixture.

## Discussion

The validation of many genotypes requires the accurate creation of specific biallelic or monoallelic combinations by the introduction of single to several nucleotide changes. Building on previous work, our results illustrate an efficient strategy for introducing defined sequence changes into PGCs using the CRISPR/Cas9 system. We show that ssODNs serve as efficient donors for precision genome editing in chicken PGCs. To the best of our knowledge, this has not been previously demonstrated for avian species or for germline stem cells. Following CRISPR/Cas9-induced DSBs, DNA repair with ssODN donors occurs through the synthesis-dependent strand annealing (SDSA) pathway^41^. We observe that HDR efficiencies with ssODN donors in chicken PGCs are up to 5-fold higher than previously reported using double stranded templates^24,25^. Previous reports show that Cas9 activity may be repressed in transcriptionally inactive targets in heterochromatin and nucleosomal DNA^68–70^. We found that HDR efficiency was unaffected by the transcriptional state of the targeted gene in PGCs (Fig. 2).

Similar to a recent report in human cells^71^, we observed using the GFP-to-BFP conversion assay that SpCas9-HF1 increases HDR levels while reducing INDEL formation (Figs 3 and 4). Since enhanced specificity Cas9 variants discriminate against targets bearing mismatches in the non-seed region of the gRNA target and prevent nuclease activation^44–46^, the higher HDR efficiency observed with SpCas9-HF1 in our results can be attributed to the high fidelity of the nuclease which proof-reads the gRNA target sequence before activating cleavage, thereby reducing re-editing of the repaired target site and leading to higher levels of HDR in the two alleles and lowering INDEL formation. As a consequence, base pair changes can be efficiently introduced into the non-seed sequence of the guide region using SpCas9-HF1 without introducing a blocking mutation into the neighbouring PAM site (Fig. 2). This enhancement in HDR accuracy by SpCas9-HF1 directly increases the efficiency of editing single alleles to generate PGCs with specific heterozygous genotypes (Figs 4 and 5C,D).

In human IPS cells, use of SpCas9-WT and ssODN donors containing appropriate CRISPR/Cas9-blocking mutations positioned in the PAM site increased HDR levels by up to a 100-fold while mutations positioned in the gRNA target sequence showed variable efficacy^35^. At this observed efficiency, one correctly edited homozygous clone was isolated for every 20 to 40 single-cell clones targeted using a single ssODN template, whereas hundreds of single cell clones were needed to isolate a biallelically edited heterozygous clone repaired using two ssODN templates^35^. In contrast to these results, using a single ssODN repair template containing CRISPR/Cas9-blocking PAM mutations and SpCas9-HF1 to introduce the *scaleless* mutation into the *FGF20* locus, we found that 4 to 5 correctly edited clones were isolated for every 10 clones screened. In contrast, we were unable to isolate a clone with precise biallelic HDR using SpCas9-WT from the number of clones that we screened which suggests that many more clones will need to be picked. In our attempt to introduce the *scaleless* mutation into one allele (*sc/*+), we were able to isolate 2 correctly edited clones containing heterozygous biallelic edits for every 10 clones screened. It must be noted that we performed single cell culturing in a growth-factors-optimised, serum-free and feeder-free culture medium^11^. In our protocol, chicken PGCs proliferate more rapidly (21-hr doubling time) than the PGCs cultured in high-serum chicken PGC medium^10^.

A major requirement for the use of SpCas9-HF1 is that the 20-nt gRNA sequence must be perfectly complementary to the genomic target to achieve high on-target editing efficiency^45^. When using the U6 promoter to drive sgRNA expression, the requirement for a 5′- G base in the sgRNA sequence limits the use of SpCas9-HF1 in targets that do not have a 5′G, and adding an extra G significantly reduces on on-target efficiency^45,72^. However, it has been shown that using alternative promoters such as the U3 promoter to express sgRNA, expressing sgRNAs from synthetic tRNA-sgRNA constructs or using hammerhead ribozyme-linked sgRNAs leads to similar levels of on-target efficiencies SpCas9-WT^72,73^.

In targeting the *FGF20* gene with SpCas9-HF1, we found that the *scaleless* 535A > T nucleotide change located 6 bp downstream of the PAM and 12 bp away from the cut site of the gRNA was incorporated biallelically at a rate of >90% in isolated HDR clones containing PAM mutations. In comparison to our results, a 12 bp cut-to-edit distance was shown to result in <20% biallelic incorporation of the edit in biallelic HDR clones in human IPS cells^35^. Surprisingly, we also observed that all INDEL clones targeted using SpCas9-WT and Sca-ssODN did not contain the 535A > T substitution or PAM mutation suggesting that these clones never underwent HDR editing event. The only CRISPR/Cas9-blocking mutation in the Sca-ssODN template is a single nucleotide substitution in the PAM (AGG → AGA) which may not be sufficient to block re-cutting of the repaired site by Cas9. It has been shown in human cells that *NGA* PAMs may have up to 40% activity in some loci^74,75^. Interestingly, we also observed that some clones contained the 535A > T substitution on only one allele while the PAM mutation was present on the two alleles suggestive of partial or incomplete HDR and has been reported by others^27,31,35^. This may be indicative of a cut-to-distance dependence mechanism in the incorporation of single nucleotide edits in chicken PGCs as previously reported in human cells and mouse zygotes^27,35^. Furthermore, we observed that Silent-ssODN was used more frequently for allelic repair when it was mixed with Sca-ssODN whereas Silent2-ssODN and Sca-ssODN were used at almost equal frequency. The absence of the distal mutation 12 bp away from the cut site in Silent-ssODN may have favoured this donor and has been previously observed in human cells^35^. Also, we did not see any evidence of template switching for allelic repair between Sca-ssODN and Silent-ssODN which has been reported to occur in human cells^41^. While we used an asymmetric ssODN donor to introduce *scaleless* 535A > T nucleotide change into *FGF20* (Fig. 5A) based on the reported ability of this template design to increase HDR^66^, we are unable to tell from our results if asymmetric repair templates are more efficient than symmetric templates in enhancing HDR in chicken PGCs and therefore may require further investigation. We also tested the use of SCR7 and L755507 to increase HDR in PGCs but we did not observe any improvement or toxicity. SCR7 and L755507 are small molecules reported to increase CRISPR-mediated HDR by inhibiting NHEJ in some cell types^34,53^. Concentration of these inhibitors may need to be optimised for PGCs. Use of these inhibitors and other reported HDR enhancers such as RS-1^76^ merit further investigation in PGCs.

Why we do observe such high HDR rates in avian PGCs using SpCas9-HF1? It is possible that many PGCs targeted with SpCas9-WT do not survive due to the induction of another round of cleavage of the HDR-edited site. Germ cells from many vertebrate species have been shown to undergo programmed cell death when exposed to reagents causing DSBs as a mechanism to protect the integrity of the germline genome^77–81^. In our experiments, we used plasmid delivery of SpCas9-WT which has been shown to have some toxicity in human embryonic stem cells compared to ribonucleoprotein (RNP) delivery^82^. In human pluripotent stem cells, it has been reported that the induction of a single DSB by SpCas9-WT is toxic even in the absence of the induction of multiple DSBs or off-target mutagenesis^83^. This toxicity is P53-dependent and the induction of P53 by Cas9 leads to apoptosis or cell cycle arrest in the G1 phase where NHEJ is predominant thereby reducing the efficiency CRISPR/Cas9 precision genome editing^83,84^. Depending on the gRNA and loci, the sustained expression of SpCas9-WT from a plasmid increases the potential for re-cleaving of HDR-edited chromosomal targets as well as off-target mutagenesis. Since the SpCas9-WT nuclease spends up to 6 hrs tightly bound to the cut ends of the DNA duplex^66^, the long residence time coupled with the high cleavage activity of SpCas9-WT probably increases the severity of genotoxic insult by preventing DNA repair, which may result in a stalled replication fork leading to cell cycle arrest or apoptosis and a decrease in overall HDR events. Indeed, inhibition of P53, a pro-apoptotic protein that is activated by DNA damage^85^, has been shown to increase the rate of HDR in human cells by preventing DNA damage response that results in apoptosis and allowing the cell cycle to progress^83,84^.

## Conclusion

Our results demonstrate possible rapid introgression of specific haplotypes into primordial germ cells. These genomic tools will allow the validation of SNP and other chromosomal changes in poultry.

## Materials and Methods

### CRISPR Plasmids

PX459 V2.0 vector^33^ was used for expression of SpCas9-WT and sgRNA. The equivalent expression cassette (HF-PX459 V2.0) for SpCas9-HF1 and sgRNA was generated by transferring the domain containing the point mutations of SpCas9-HF1 into the coding sequence of SpCas9-WT in PX459 V2.0. A 1.4 kb region of the coding sequence of SpCas9-WT in PX459 V2.0 was excised using ApaI and PmlI restriction digest and replaced with a homologous 1.4 kb overlapping PCR fragment (Left primer, 5′-TTCCGCATCCCCTACTACGTGGGCCCCCTGGCCCGAGGGAACTCTC-3′ and right primer 5′-TCCGGGAGTCCAGGATCTGTGCCACATGCTTTGTGATGGCGCGGGTTTC-3′) from the coding sequence of SpCas9-HF1 in VP12 vector^45^ using NEBuilder® HiFi DNA Assembly (New England Biolabs). PX459 V2.0 was a gift from Feng Zhang (Addgene plasmid # 62988) while VP12 was a gift from Keith Joung (Addgene plasmid # 72247).

### gRNA design and CRISPR/Cas9 vector construction

gRNA sequences were selected using CHOPCHOP gRNA design web tool (http://chopchop.cbu.uib.no/)^86,87^ and MIT gRNA design web tool (http://crispr.mit.edu/) except where described otherwise. gRNA oligonucleotides were synthesized by Invitrogen and inserted into PX459 V2.0 and HF-PX459 V2.0 vectors using methods previously described in^33^. gRNA sequences are listed in Supplementary Table S1.

### ssODN donors

ssODN donors were Ultramer® DNA Oligonucleotides synthesized by Integrated DNA Technologies (IDT). Donors were used at a concentration of 10 µM for transfections. See Supplementary Table S2 for sequences of ssODN donors.

### Animal usage

Fertile eggs were obtained from a flock of commercial Hyline layer hens maintained at the Roslin Institute. The GFP^+/+^ germ cells used in the experiments shown in Figs 3 and 4 were obtained by crossing the Roslin Green (ubiquitous GFP) line of transgenic chicken^88^ to produce homozygous fertile eggs for PGC derivations. Commercial and transgenic chicken lines were maintained and bred under UK Home Office License. All experiments were performed in accordance with relevant UK Home Office guidelines and regulations. The experimental protocol and studies were reviewed by the Roslin Institute Animal Welfare and Ethical Review Board (AWERB) Committee.

### PGC culture

PGC lines were derived and cultured in FAOT medium as described in Whyte *et al*.^11^. Briefly, fertile eggs from Hyline layer lines or the Roslin Green line of transgenic chickens^88^ were incubated for 2.5 days and then 1 μl of embryonic blood was taken from the vasculature of stage 16 HH chick embryos^89^ and placed into FAOT medium. FAOT medium contains 1 × B-27 supplement (Thermo Fisher Scientific), 2.0 mM GlutaMax (Thermo Fisher Scientific), 1 × non-essential amino acids (Thermo Fisher Scientific), 1 × EmbryoMax nucleosides (Merck Millipore), 0.1 mM β-mercaptoethanol (Thermo Fisher Scientific), 0.2% ovalbumin (Sigma), 1.2 mM pyruvate (Thermo Fisher Scientific), 0.15 mM CaCl_2_, 0.01% sodium heparin (Sigma), 4 ng/ml FGF2 (R&D Systems), 25 ng/ml activin A (Peprotech) and 5 µg/ml ovotransferrin (Sigma) in Avian Knockout DMEM (osmolality: 250 mOsmol/kg, 12.0 mM glucose, calcium chloride free; Thermo Fisher Scientific, a custom modification of Knockout DMEM). PGC lines were expanded to 2.4 × 10^5^ cells in 5 weeks before use in targeting experiments. GFP-PGCs were derived from transgenic chickens created by lentiviral methods^88^ and maintained at the National Avian Research Facility, Midlothian, UK.

### PGC transfection

200,000 PGCs were transfected with 1.5 µg of CRISPR vector and 0.4 µg of ssODN donor using Lipofectamine 2000 transfection reagent (Thermo Fisher Scientific). Cells were washed in Optimem I (Thermo Fisher Scientific) and incubated in suspension in Optimem I mixed with DNA and transfection reagent for 6 h. PGCs were then centrifuged and resuspended in FAOT medium. PGCs were treated with 0.6 µg/ml puromycin 24 h post-transfection for 48 h to enrich for transfected cells and then expanded in culture for two weeks to eliminate transient CRISPR expression and then used to isolate genomic DNA to measure targeting efficiencies or isolate single cells. To test NHEJ inhibitors, PGCs were treated with SCR7 (Sigma-Aldrich) at a concentration of 1 mM or L755507 (Sigma-Aldrich) at a concentration of 5 µM for 24 h after transfection followed by puromycin treatment for 48 h.

### Isolation of single-cell clonal populations

PGCs were seeded manually or sorted using a FACSAria III (BD Biosciences) into 96-well plates at 1 cell per well in 110 µL FAOT medium and cultured for 2 weeks. Once cell density reached 30–50%, PGCs were transferred into a 48-well plate and subsequently into a 24-well plate for further expansion to isolate genomic DNA for analysis of mutation genotype.

### Genomic DNA isolation and PCR amplification of target region

Genomic DNA was extracted from PGCs using QIAMP Micro Kit (Qiagen) according to the manufacturer’s instruction. Specific primers for PCR amplification of sgRNA target sites were designed using primer3 software (http://primer3.ut.ee/)^90,91^. Primers were designed to anneal outside the homology arms of HDR templates. List of primer sequences are listed in Supplementary Table S3. All PCR amplifications were performed using 100 ng of genomic DNA and Q5® Hot Start High-Fidelity DNA polymerase (NEB) or Phusion® High-Fidelity DNA polymerase (NEB) according to the manufacturer’s protocol. Primer annealing temperatures were calculated using the online NEB Tm calculator (https://tmcalculator.neb.com).

### Analysis of gRNA targeting efficiency by T7 endonuclease I mismatch assay

T7 endonuclease I assay was performed by treating 200 ng of PCR DNA with 10 units of T7 endonuclease I in NEBuffer™ 2 buffer and incubated according to the manufacturer’s instruction (NEB). The digestion products were resolved on 1% agarose gel containing Gelred® nucleic acid gel stain (Biotium) and ImageJ (https://imagej.net) was used to quantitate band intensities. The targeting efficiency was calculated using the equation 100 × (1 − (1 − fraction cleaved)1/2)^92^.

### HDR quantification by *Eco*RI restriction digestion

Restriction site sequences were incorporated into the target site to quantify HDR. All restriction enzyme digestion reactions were performed by treating 200 ng of PCR DNA with 10 units of restriction enzyme (*EcoRI-HF* (NEB #R3101S)*, BamHI-HF* (NEB #R3136S)*, AfeI* (NEB #R0652S), *AatII* (NEB #R0117S)) in CutSmart buffer (NEB) incubated at 37 °C for 1 h. The digestion products were resolved on 1% agarose gel containing Gelred® nucleic acid gel stain (Biotium). ImageJ software was used to quantitate band intensities. The percentage of HDR was calculated as described in^51^ using the equation (b + c/a + b + c) × 100, where ‘a’ is the band intensity of uncleaved DNA substrate and ‘b’ and ‘c’ are the cleavage products.

### HDR and INDEL quantification by flow cytometry

Live cells were gated and then GFP and BFP fluorescence was detected using a Fortessa X20 (BD Biosciences). Cytometry data was analysed using FlowJo® V7.0 (FlowJo, LLC).

### DNA sequencing and bioinformatic analysis

PCR products were directly sequenced to analyse mutation genotypes of single cell clones using PCR primers or PCR products were cloned into pGEM-T Easy vector (Promega #A137A) and sequenced using T7 promoter forward primer by Sanger sequencing. Sequencing data was analysed and viewed using SeqMan Pro 13 (Lasergene 13, DNASTAR) and FinchTV 1.4.0 (Geospiza, Inc.). PCR Sanger sequencing traces were analysed with the TIDE analysis web tool (https://tide.deskgen.com/) to detect INDELs in a population.

## Electronic supplementary material

Supplementary Figures and Tables

## Data Availability

Plasmid HF-PX459 V2.0 will be available from Addgene. Data sharing is not applicable to this article as no datasets were generated or analysed during the current study.

## References

[1] Lillico SG, McGrew MJ, Sherman A, Sang HM (2005). Transgenic chickens as bioreactors for protein-based drugs. Drug Discov. Today.

[2] Scott BB, Velho TA, Sim S, Lois C (2010). Applications of avian transgenesis. ILAR J..

[3] FAO. FAOSTAT- Livestock primary data. Available at: http://www.fao.org/faostat/en/#data/QL. (Accessed: 6th May 2018) (2016).

[4] Brinster RL, Chen HY, Trumbauer ME, Yagle MK, Palmiter RD (1985). Factors affecting the efficiency of introducing foreign DNA into mice by microinjecting eggs. Proc. Natl. Acad. Sci..

[5] Thompson S, Clarke AR, Pow AM, Hooper ML, Melton DW (1989). Germ line transmission and expression of a corrected HPRT gene produced by gene targeting in embryonic stem cells. Cell.

[6] Campbell KHS, McWhir J, Ritchie WA, Wilmut I (1996). Sheep cloned by nuclear transfer from a cultured cell line. Nature.

[7] Schnieke AE (1997). Human Factor IX Transgenic Sheep Produced by Transfer of Nuclei from Transfected Fetal Fibroblasts. Science (80-.)..

[8] Sang HM, Perry MM (1989). Episomal replication of cloned DNA injected into the fertilised ovum of the hen, Gallus domesticus. Mol. Reprod. Dev..

[9] van de Lavoir M-C (2006). High-grade transgenic somatic chimeras from chicken embryonic stem cells. Mech. Dev..

[10] van de Lavoir M-C (2006). Germline transmission of genetically modified primordial germ cells. Nature.

[11] Whyte J (2015). FGF, Insulin, and SMAD Signaling Cooperate for Avian Primordial Germ Cell Self-Renewal. Stem Cell Reports.

[12] Woodcock Mark E., Idoko-Akoh Alewo, McGrew Michael J. (2017). Gene editing in birds takes flight. Mammalian Genome.

[13] Schusser B (2013). Immunoglobulin knockout chickens via efficient homologous recombination in primordial germ cells. Proc. Natl. Acad. Sci. USA.

[14] Jasin M (1996). Genetic manipulation of genomes with rare-cutting endonucleases. Trends Genet..

[15] Gaj T, Gersbach CA, Barbas CF (2013). ZFN, TALEN, and CRISPR/Cas-based methods for genome engineering. Trends Biotechnol..

[16] Rothkamm K, Krüger I, Thompson LH, Kru I, Lo M (2003). Pathways of DNA Double-Strand Break Repair during the Mammalian Cell Cycle Pathways of DNA Double-Strand Break Repair during the Mammalian Cell Cycle. Mol. Cell. Biol..

[17] Pardo B, Gómez-González B, Aguilera A (2009). DNA Repair in Mammalian Cells. Cell. Mol. Life Sci..

[18] Shrivastav M, De Haro LP, Nickoloff JA, Haro LP (2008). De. Regulation of DNA double-strand break repair pathway choice. Cell Res..

[19] Chang HHY, Pannunzio NR, Adachi N, Lieber MR (2017). Non-homologous DNA end joining and alternative pathways to double-strand break repair. Nat. Rev. Mol. Cell Biol..

[20] Li X, Heyer W-D (2008). Homologous recombination in DNA repair and DNA damage tolerance. Cell Res.

[21] Orthwein A (2015). A mechanism for the suppression of homologous recombination in G1 cells. Nature.

[22] Park TS, Lee HJ, Kim KH, Kim J-S, Han JY (2014). Targeted gene knockout in chickens mediated by TALENs. Proc. Natl. Acad. Sci. USA.

[23] Oishi I, Yoshii K, Miyahara D, Kagami H, Tagami T (2016). Targeted mutagenesis in chicken using CRISPR/Cas9 system. Sci. Rep..

[24] Dimitrov L (2016). Germline gene editing in chickens by efficient crispr-mediated homologous recombination in primordial germ cells. PLoS One.

[25] Taylor L (2017). Efficient TALEN-mediated gene targeting of chicken primordial germ cells. Development.

[26] Armstrong GAB (2016). Homology Directed Knockin of Point Mutations in the Zebrafish tardbp and fus Genes in ALS Using the CRISPR/Cas9 System. PLoS One.

[27] Inui M (2014). Rapid generation of mouse models with defined point mutations by the CRISPR/Cas9 system. Sci. Rep..

[28] Kistler KE, Vosshall LB, Matthews BJ (2015). Genome Engineering with CRISPR-Cas9 in the Mosquito Aedes aegypti. Cell Rep..

[29] Long C (2014). Prevention of muscular dystrophy in mice by CRISPR/Cas9–mediated editing of germline DNA. Science (80-.)..

[30] Port F, Chen H-M, Lee T, Bullock SL (2014). Optimized CRISPR/Cas tools for efficient germline and somatic genome engineering in *Drosophila* Proc. Natl. Acad. Sci..

[31] Wang K (2016). Efficient Generation of Orthologous Point Mutations in Pigs via CRISPR-assisted ssODN-mediated Homology-directed Repair. Mol. Ther. - Nucleic Acids.

[32] Xiaoyang Z (2015). Efficient Generation of Gene‐Modified Pigs Harboring Precise Orthologous Human Mutation via CRISPR/Cas9‐Induced Homology‐Directed Repair in Zygotes. Hum. Mutat..

[33] Ran FA (2013). Genome engineering using the CRISPR-Cas9 system. Nat. Protoc..

[34] Yu C (2015). Small Molecules Enhance CRISPR Genome Editing in Pluripotent Stem Cells. Cell Stem Cell.

[35] Paquet D (2016). Efficient introduction of specific homozygous and heterozygous mutations using CRISPR/Cas9. Nature.

[36] Yang L (2013). Optimization of scarless human stem cell genome editing. Nucleic Acids Res..

[37] Hwang WY (2013). Efficient genome editing in zebrafish using a CRISPR-Cas system. Nat. Biotechnol..

[38] Wang L (2017). Enhancing Targeted Genomic DNA Editing in Chicken Cells Using the CRISPR/Cas9 System. PLoS One.

[39] Olsen PA, Solhaug A, Booth JA, Gelazauskaite M, Krauss S (2009). Cellular responses to targeted genomic sequence modification using single-stranded oligonucleotides and zinc-finger nucleases. DNA Repair (Amst)..

[40] Rios X (2012). Stable Gene Targeting in Human Cells Using Single-Strand Oligonucleotides with Modified Bases. PLoS One.

[41] Paix A (2017). Precision genome editing using synthesis-dependent repair of Cas9-induced DNA breaks. Proc. Natl. Acad. Sci..

[42] Bialk, P. *et al*. Analyses of point mutation repair and allelic heterogeneity generated by CRISPR/Cas9 and single-stranded DNAoligonucleotides. *Sci. Rep*. 10.1038/srep32681 (2016). 10.1038/srep32681 PMC5016854 27609304

[43] Merkle Florian T., Neuhausser Werner M., Santos David, Valen Eivind, Gagnon James A., Maas Kristi, Sandoe Jackson, Schier Alexander F., Eggan Kevin (2015). Efficient CRISPR-Cas9-Mediated Generation of Knockin Human Pluripotent Stem Cells Lacking Undesired Mutations at the Targeted Locus. Cell Reports.

[44] Chen JS (2017). Enhanced proofreading governs CRISPR–Cas9 targeting accuracy. Nature.

[45] Kleinstiver BP (2016). High-fidelity CRISPR–Cas9 nucleases with no detectable genome-wide off-target effects. Nature.

[46] Slaymaker IM (2016). Rationally engineered Cas9 nucleases with improved specificity. Science.

[47] Glaser, A., McColl, B. & Vadolas, J. GFP to BFP Conversion: A Versatile Assay for the Quantification of CRISPR/Cas9-mediated Genome Editing. *Mol. Ther. - Nucleic Acids* **5** (2017). 10.1038/mtna.2016.48 PMC5330940 27404719

[48] Wells KL (2012). Genome-wide SNP scan of pooled DNA reveals nonsense mutation in FGF20 in the scaleless line of featherless chickens. BMC Genomics.

[49] Cahaner A (2008). Effects of the Genetically Reduced Feather Coverage in Naked Neck and Featherless Broilers on Their Performance Under Hot Conditions. Poult. Sci..

[50] Azoulay Y (2011). The viability and performance under hot conditions of featherless broilers versus fully feathered broilers. Poult Sci.

[51] Lin S, Staahl BT, Alla RK, Doudna JA (2014). Enhanced homology-directed human genome engineering by controlled timing of CRISPR/Cas9 delivery. Elife.

[52] Oji A (2016). CRISPR/Cas9 mediated genome editing in ES cells and its application for chimeric analysis in mice. Sci. Rep..

[53] Maruyama T (2015). Increasing the efficiency of precise genome editing with CRISPR-Cas9 by inhibition of nonhomologous end joining. Nat. Biotechnol..

[54] Jiang W, Bikard D, Cox D, Zhang F, Marraffini LA (2013). RNA-guided editing of bacterial genomes using CRISPR-Cas systems. Nat. Biotechnol..

[55] Zheng, T. *et al*. Profiling single-guide RNA specificity reveals a mismatch sensitive core sequence. *Sci. Rep*. **7** (2017). 10.1038/srep40638 PMC5241822 28098181

[56] Semenova E (2011). Interference by clustered regularly interspaced short palindromic repeat (CRISPR) RNA is governed by a seed sequence. Proc. Natl. Acad. Sci..

[57] Jinek M (2012). A programmable dual-RNA-guided DNA endonuclease in adaptive bacterial immunity. Science (New York, N.Y.).

[58] Hsu PD (2013). DNA targeting specificity of RNA-guided Cas9 nucleases. Nat. Biotechnol..

[59] Fu Y (2013). High-frequency off-target mutagenesis induced by CRISPR-Cas nucleases in human cells. Nat. Biotechnol..

[60] Cho SW (2014). Analysis of off-target effects of CRISPR/Cas-derived RNA-guided endonucleases and nickases. Genome Res..

[61] Gao Z, Harwig A, Berkhout B, Herrera-Carrillo E (2017). Mutation of nucleotides around the+1 position of type 3 polymerase III promoters: The effect on transcriptional activity and start site usage. Transcription.

[62] Howden Sara E., McColl Bradley, Glaser Astrid, Vadolas Jim, Petrou Steven, Little Melissa H., Elefanty Andrew G., Stanley Edouard G. (2016). A Cas9 Variant for Efficient Generation of Indel-Free Knockin or Gene-Corrected Human Pluripotent Stem Cells. Stem Cell Reports.

[63] Brinkman EK, Chen T, Amendola M, van Steensel B (2014). Easy quantitative assessment of genome editing by sequence trace decomposition. Nucleic Acids Res..

[64] Wang H (2013). One-Step Generation of Mice Carrying Mutations in Multiple Genes by CRISPR/Cas-Mediated Genome Engineering. Cell.

[65] Liang X, Potter J, Kumar S, Ravinder N, Chesnut JD (2017). Enhanced CRISPR/Cas9-mediated precise genome editing by improved design and delivery of gRNA, Cas9 nuclease, and donor DNA. J. Biotechnol..

[66] Richardson CD, Ray GJ, DeWitt MA, Curie GL, Corn JE (2016). Enhancing homology-directed genome editing by catalytically active and inactive CRISPR-Cas9 using asymmetric donorDNA. Nat Biotech.

[67] Sentmanat MF, Peters ST, Florian CP, Connelly JP, Pruett-Miller SM (2018). A Survey of Validation Strategies for CRISPR-Cas9 Editing. Sci. Rep..

[68] Knight SC (2015). Dynamics of CRISPR-Cas9 genome interrogation in living cells. Science (80-.)..

[69] Chen X (2016). Probing the impact of chromatin conformation on genome editing tools. Nucleic Acids Res..

[70] Horlbeck MA (2016). Nucleosomes impede Cas9 access to DNA *in vivo* and *in vitro*. Elife.

[71] Kato-Inui, T., Takahashi, G., Hsu, S. & Miyaoka, Y. Clustered regularly interspaced short palindromic repeats (CRISPR)/CRISPR-associated protein 9 with improved proof-reading enhances homology-directed repair. *Nucleic Acids Res*. gky264-gky264 (2018). 10.1093/nar/gky264 PMC5961419 29672770

[72] Zhang D (2017). Perfectly matched 20-nucleotide guide RNA sequences enable robust genome editing using high-fidelity SpCas9 nucleases. Genome Biol..

[73] Kim S, Bae T, Hwang J, Kim J-S (2017). Rescue of high-specificity Cas9 variants using sgRNAs with matched 5′ nucleotides. Genome Biol..

[74] Kleinstiver BP (2015). Engineered CRISPR-Cas9 nucleases with altered PAM specificities. Nature.

[75] Zhang Y (2014). Comparison of non-canonical PAMs for CRISPR/Cas9-mediated DNA cleavage in human cells. Sci. Rep..

[76] Song J (2016). RS-1 enhances CRISPR/Cas9- and TALEN-mediated knock-in efficiency. Nat. Commun..

[77] Hou M (2005). Doxorubicin Induces Apoptosis in Germ Line Stem Cells in the Immature Rat Testis and Amifostine Cannot Protect against This Cytotoxicity. Cancer Res..

[78] Olsen A-K, Lindeman B, Wiger R, Duale N, Brunborg G (2005). How do male germ cells handle DNA damage?. Toxicol. Appl. Pharmacol..

[79] Liu G (2006). Effect of Low-Level Radiation on the Death of Male Germ Cells. Radiat. Res..

[80] Xu G, Vogel KS, McMahan CA, Herbert DC, Walter CA (2010). BAX and Tumor Suppressor TRP53 Are Important in Regulating Mutagenesis in Spermatogenic Cells in Mice. Biol. Reprod..

[81] Habas K, Anderson D, Brinkworth MH (2017). Germ cell responses to doxorubicin exposure *in vitro*. Toxicol. Lett..

[82] Kim S, Kim D, Cho SW, Kim J, Kim J-S (2014). Highly efficient RNA-guided genome editing in human cells via delivery of purified Cas9 ribonucleoproteins. Genome Res..

[83] Ihry Robert J., Worringer Kathleen A., Salick Max R., Frias Elizabeth, Ho Daniel, Theriault Kraig, Kommineni Sravya, Chen Julie, Sondey Marie, Ye Chaoyang, Randhawa Ranjit, Kulkarni Tripti, Yang Zinger, McAllister Gregory, Russ Carsten, Reece-Hoyes John, Forrester William, Hoffman Gregory R., Dolmetsch Ricardo, Kaykas Ajamete (2018). p53 inhibits CRISPR–Cas9 engineering in human pluripotent stem cells. Nature Medicine.

[84] Haapaniemi Emma, Botla Sandeep, Persson Jenna, Schmierer Bernhard, Taipale Jussi (2018). CRISPR–Cas9 genome editing induces a p53-mediated DNA damage response. Nature Medicine.

[85] Fridman JS, Lowe SW (2003). Control of apoptosis by p53. Oncogene.

[86] Labun K, Montague TG, Gagnon JA, Thyme SB, Valen E (2016). CHOPCHOPv2: a web tool for the next generation of CRISPR genome engineering. Nucleic Acids Res..

[87] Montague TG, Cruz JM, Gagnon JA, Church GM, Valen E (2014). CHOPCHOP: a CRISPR/Cas9 and TALEN web tool for genome editing. Nucleic Acids Res..

[88] McGrew MJ (2008). Localised axial progenitor cell populations in the avian tail bud are not committed to a posterior Hox identity. Development.

[89] Hamburger V, Hamilton HL (1992). A series of normal stages in the development of the chick embryo. Dev. Dyn..

[90] Untergasser A (2012). Primer3—new capabilities and interfaces. Nucleic Acids Res..

[91] Koressaar T, Remm M (2007). Enhancements and modifications of primer design program Primer3. Bioinformatics.

[92] Guschin Dmitry Y., Waite Adam J., Katibah George E., Miller Jeffrey C., Holmes Michael C., Rebar Edward J. (2010). A Rapid and General Assay for Monitoring Endogenous Gene Modification. Methods in Molecular Biology.

