# Supplementary material for: High fidelity CRISPR/Cas9 increases precise monoallelic and biallelic editing events in primordial germ cells
Source: Sci Rep. 2018 Oct 11;8:15126. doi: 10.1038/s41598-018-33244-x (PMC6181960; doi:10.1038/s41598-018-33244-x)
Supplement: Supplementary file 1 — Supplementary Figures and Tables [file 41598_2018_33244_MOESM1_ESM.docx]

**SUPPLEMENTARY INFORMATION**

**High fidelity CRISPR/Cas9 increases precise monoallelic and biallelic editing events in primordial germ cells**

Alewo Idoko-Akoh, Lorna Taylor, Helen M. Sang and Michael J. McGrew

**
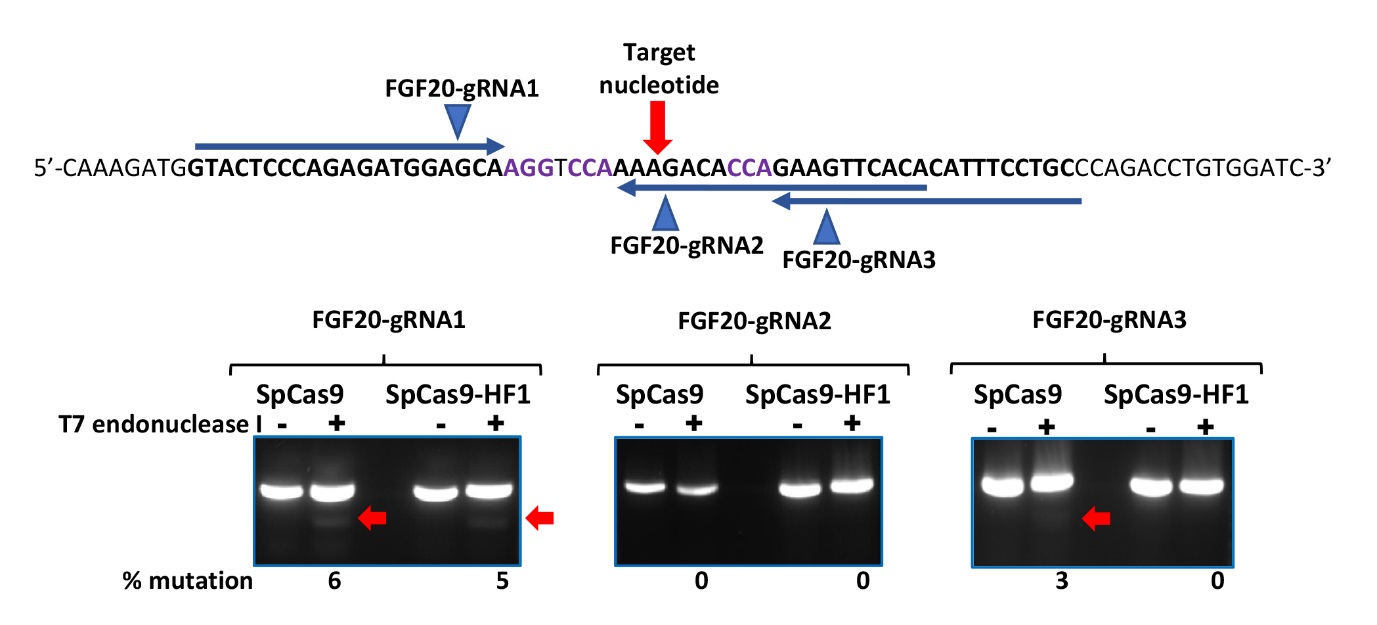
**

**Figure S1.** Analysis of gRNA activity in FGF20 using T7 endonuclease I mismatch assay. gRNA sequences are highlighted in blue. PAMs are highlighted in purple. Blue arrowheads indicate Cas9 cleavage site. Red arrows point to cleaved PCR substrates (See Figure S8 for uncropped images).

**
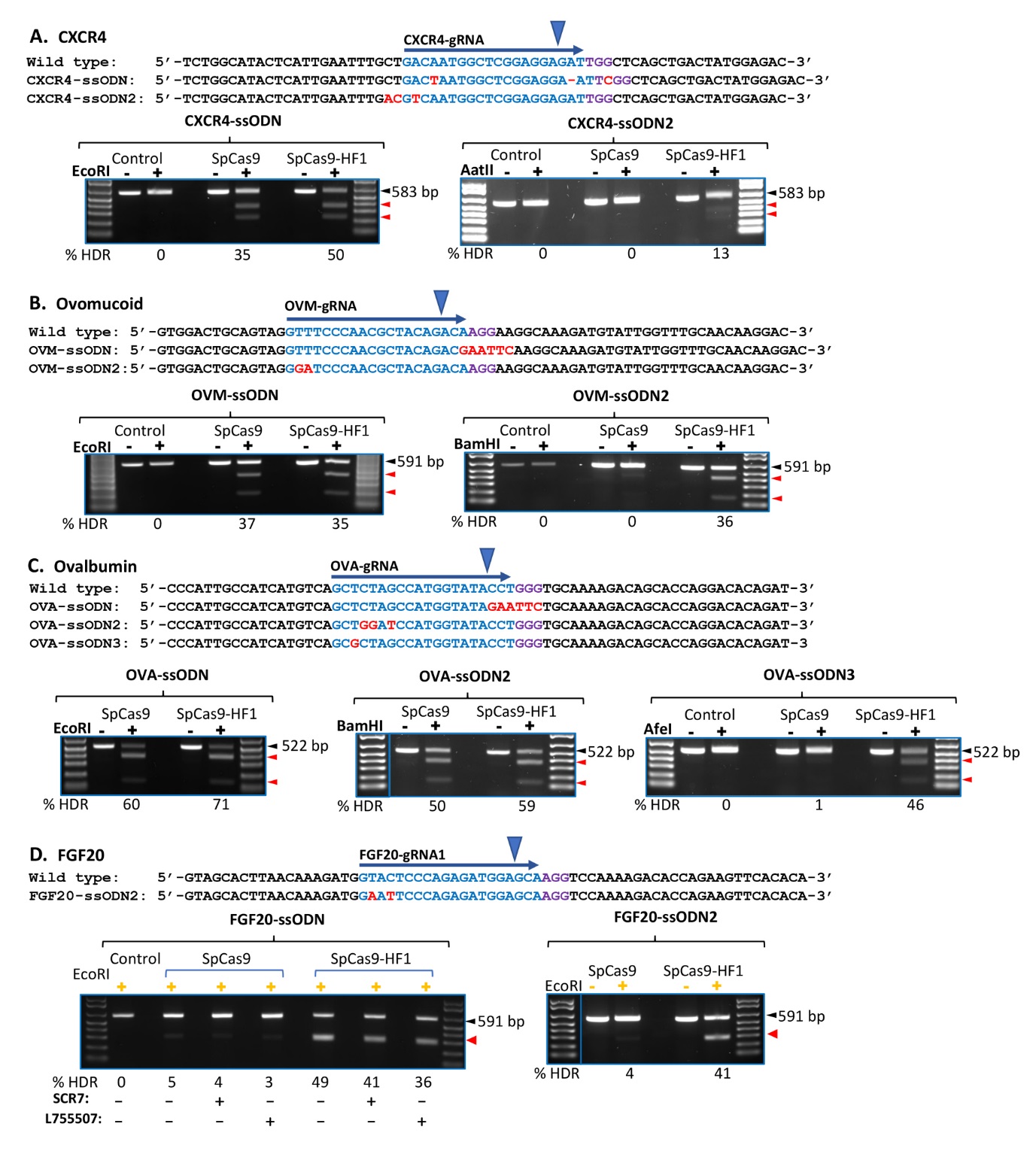
**

**Figure S2.** Second independent experiments showing efficient oligonucleotide-template HDR editing across multiple loci using SpCas9-HF1. gRNA sequences are highlighted in blue. PAMs are highlighted in purple. Blue arrowheads indicate Cas9 cleavage site. Black arrowheads indicate undigested PCR substrate. Red arrowheads indicate digested PCR products. (**-**) untreated substrate. (**+**) treated substrate. (See Figure S10 for uncropped images)


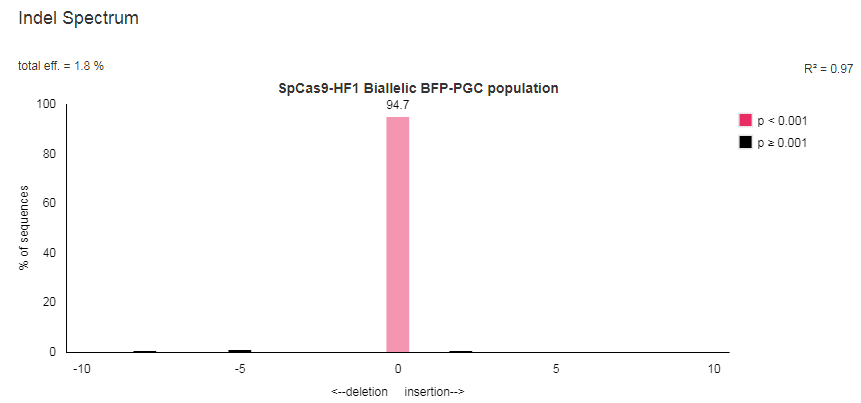


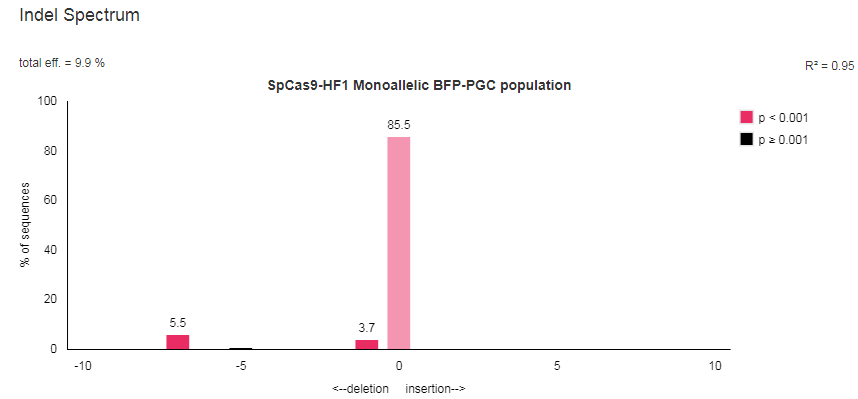


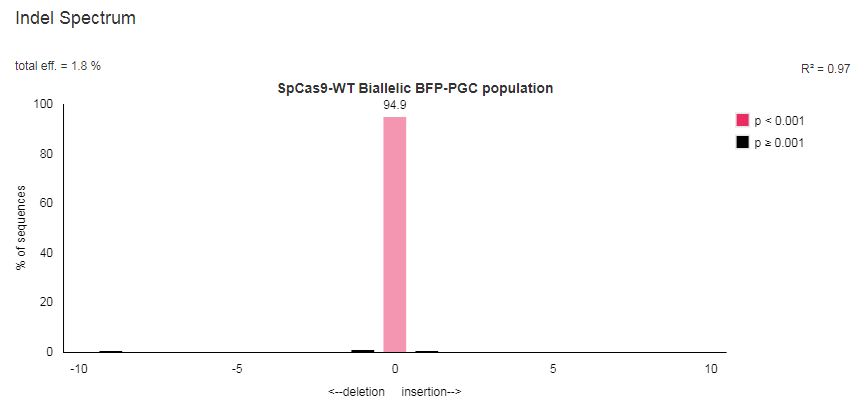


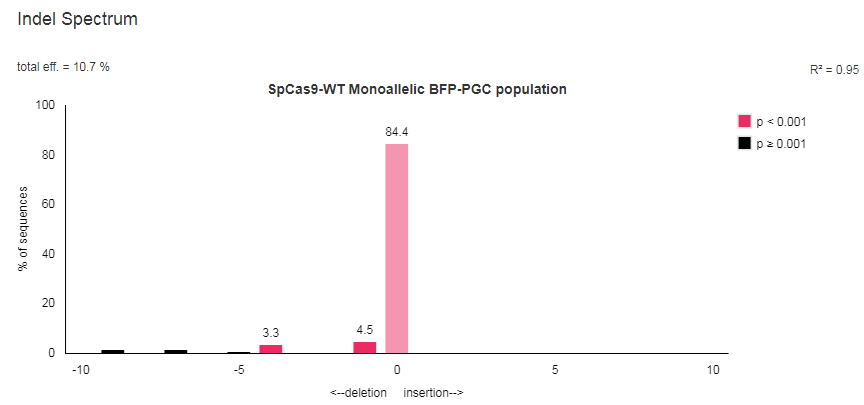


**Figure S3.** Quantification of INDEL events in isolated BFP-PGC populations (Fig. 3C) using TIDE analysis of Sanger sequencing trace files.


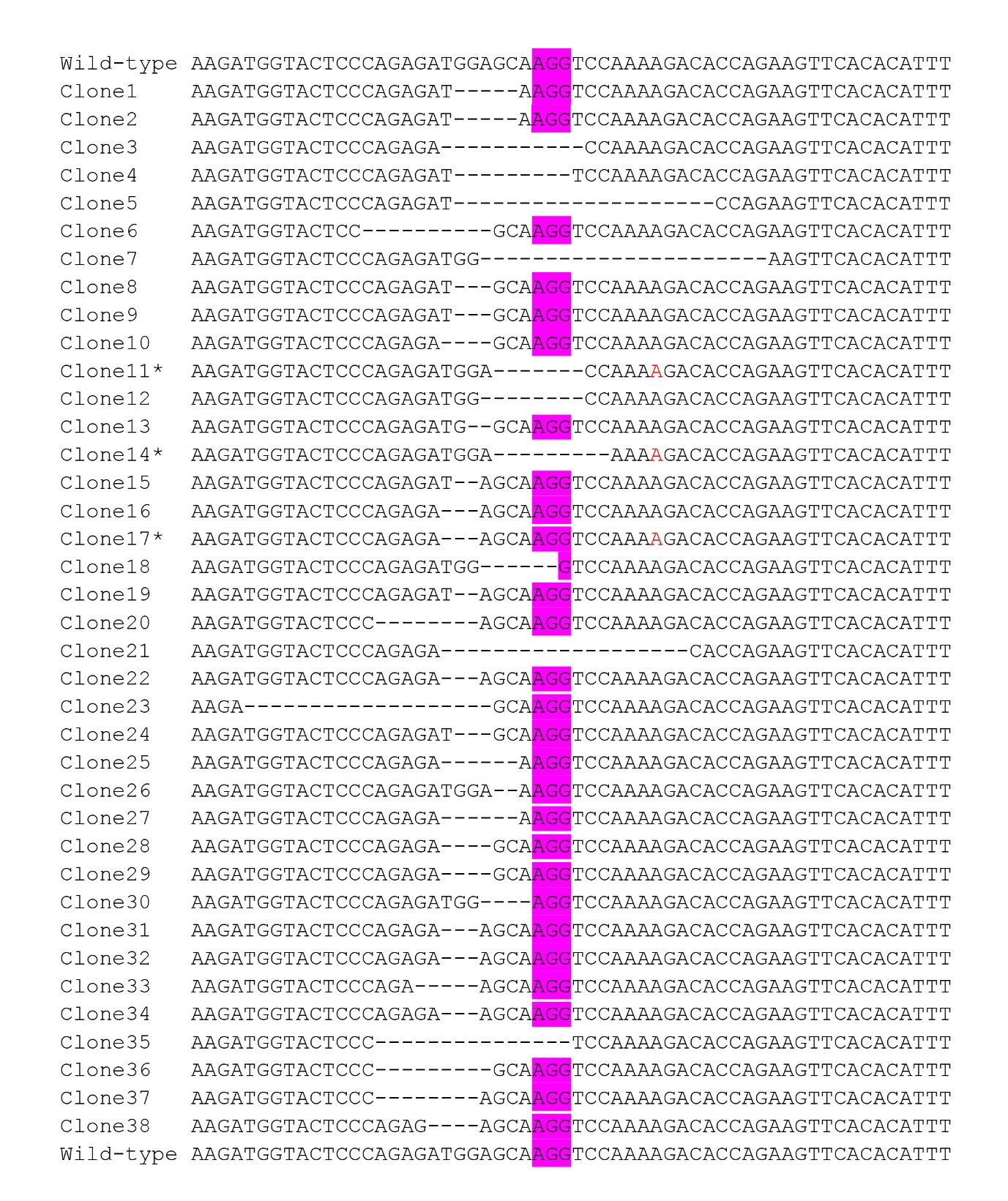


**Figure S4.** Alignment of sequences from single-cell clones targeted with SpCas9-WT and Sca-ssODN. *Sca/INDEL clones** - refers clones with monoallelic HDR


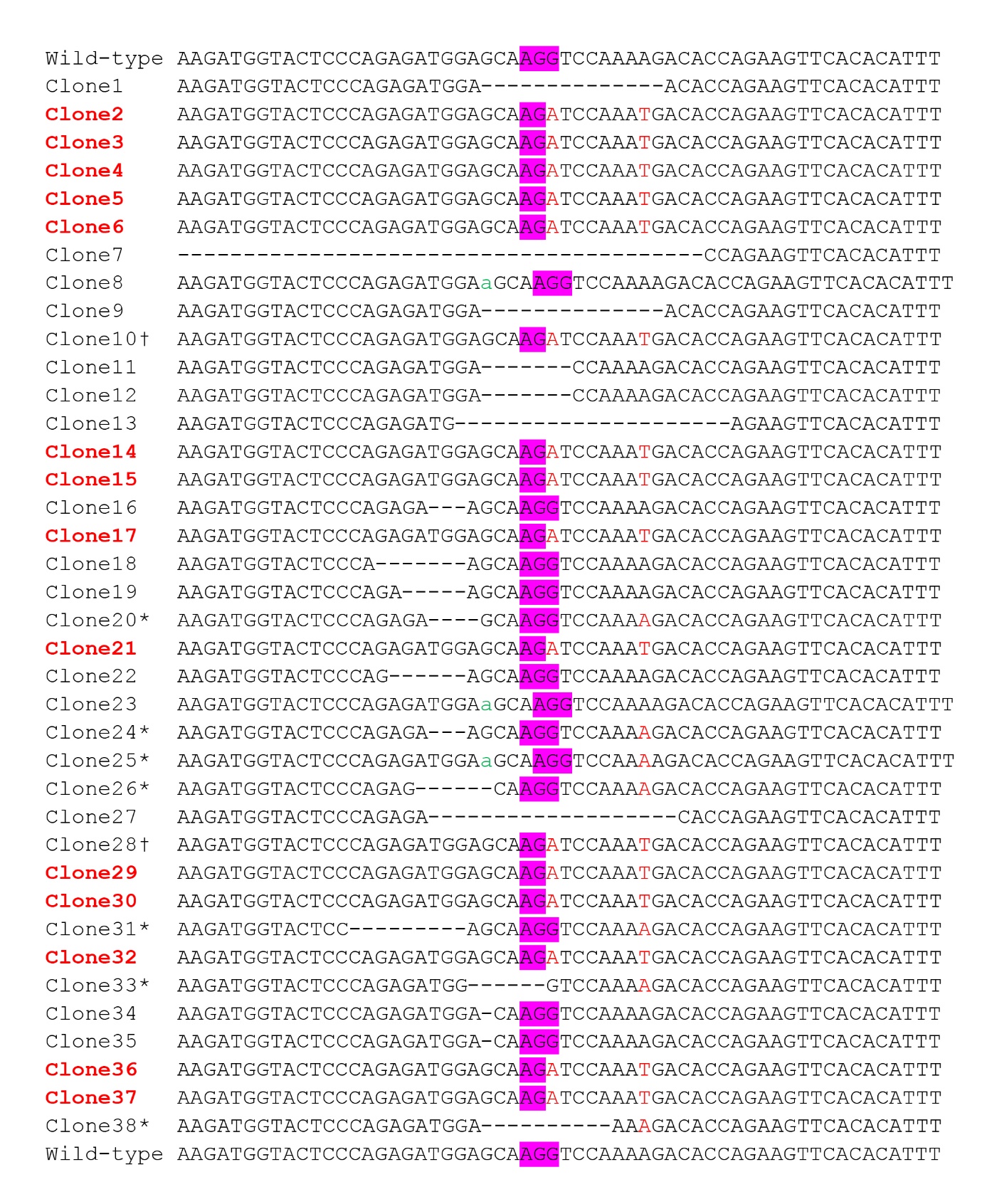


**Figure S5.** Alignment of sequences from single-cell clones targeted with SpCas9-HF1 and Sca-ssODN. Sca/INDEL clones*-refers to clones with monoallelic HDR. Sca/Sca*WT†-refers to clones with biallelic PAM mutation and monoallelic *scaleless* 535A > T substitution. Sca/Sca clones with biallelic PAM mutation and biallelic *scaleless* 535A > T substitution are highlighted in red.


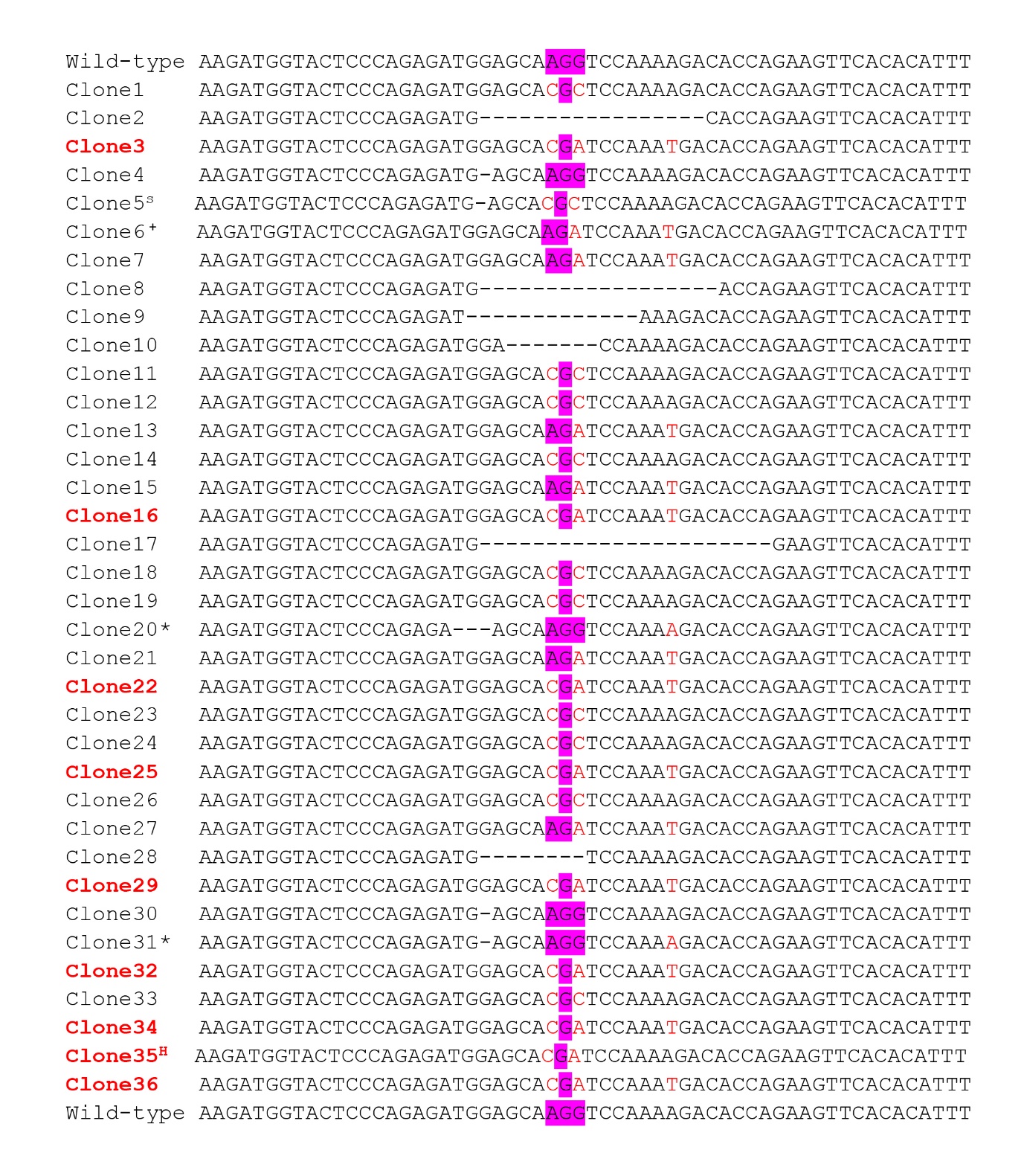


**Figure S6.** Alignment of sequences from single-cell clones targeted with SpCas9-HF1 and mixture of Sca-ssODN and Silent-ssODN. *-refers to *Sca/*INDEL clones with monoallelic HDR using Sca-ssODN. †-refers to Sca/Sca*WT clones with biallelic PAM mutation and monoallelic *scaleless* 535A > T substitution. ^s^-refers to Silent/INDEL clones with monoallelic HDR using Silent-ssODN. Sca/Silent clones with biallelic PAM mutation and monoallelic *scaleless* 535A > T substitution are highlighted in red. ^H^-refers to Sca/Silent clone with biallelic PAM mutation but no *scaleless* 535A > T substitution.


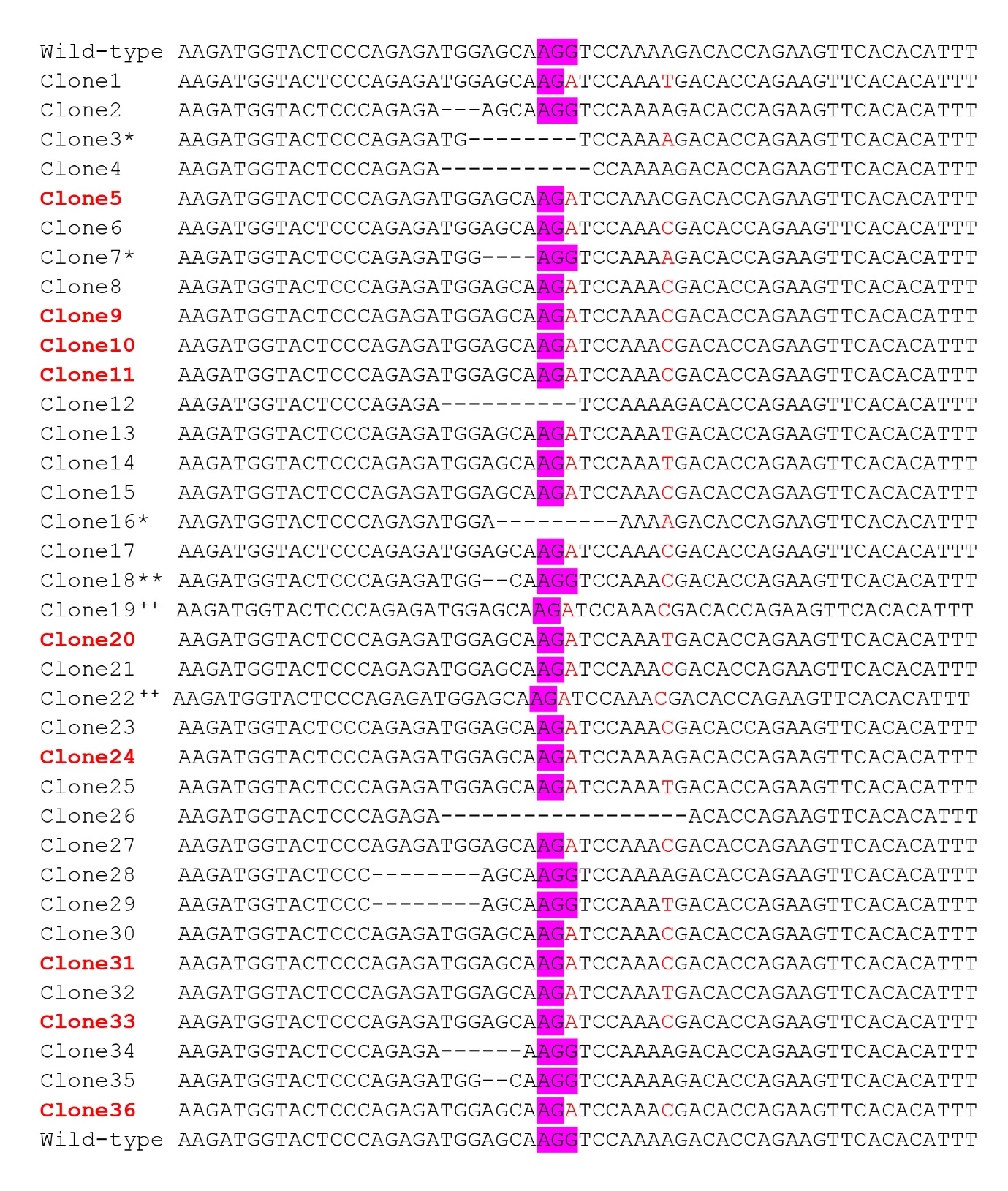


**Figure S7.** Alignment of sequences from single-cell clones targeted with SpCas9-HF1 and mixture of Sca-ssODN and Silent2-ssODN. *-refers to Sca/INDEL clones with monoallelic HDR using Sca-ssODN. †-refers to Sca/Sca*WT clones with biallelic PAM mutation and monoallelic *scaleless* 535A > T substitution. **-refers to Silent2/INDEL clones with monoallelic HDR using Silent2-ssODN. ††-refers to Silent2/Silent2 clones with biallelic PAM mutation and monoallelic *scaleless* 535A > C substitution. Sca/Silent2 clones with biallelic PAM mutation and monoallelic *scaleless* 535A > C/T substitution are highlighted in red.


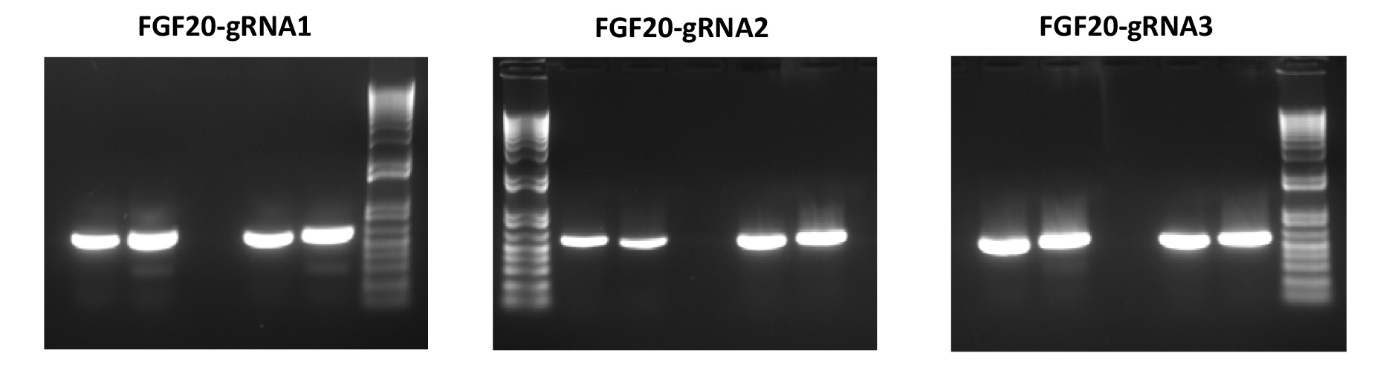


**Figure S8**. Uncropped gel images for Figure S1.


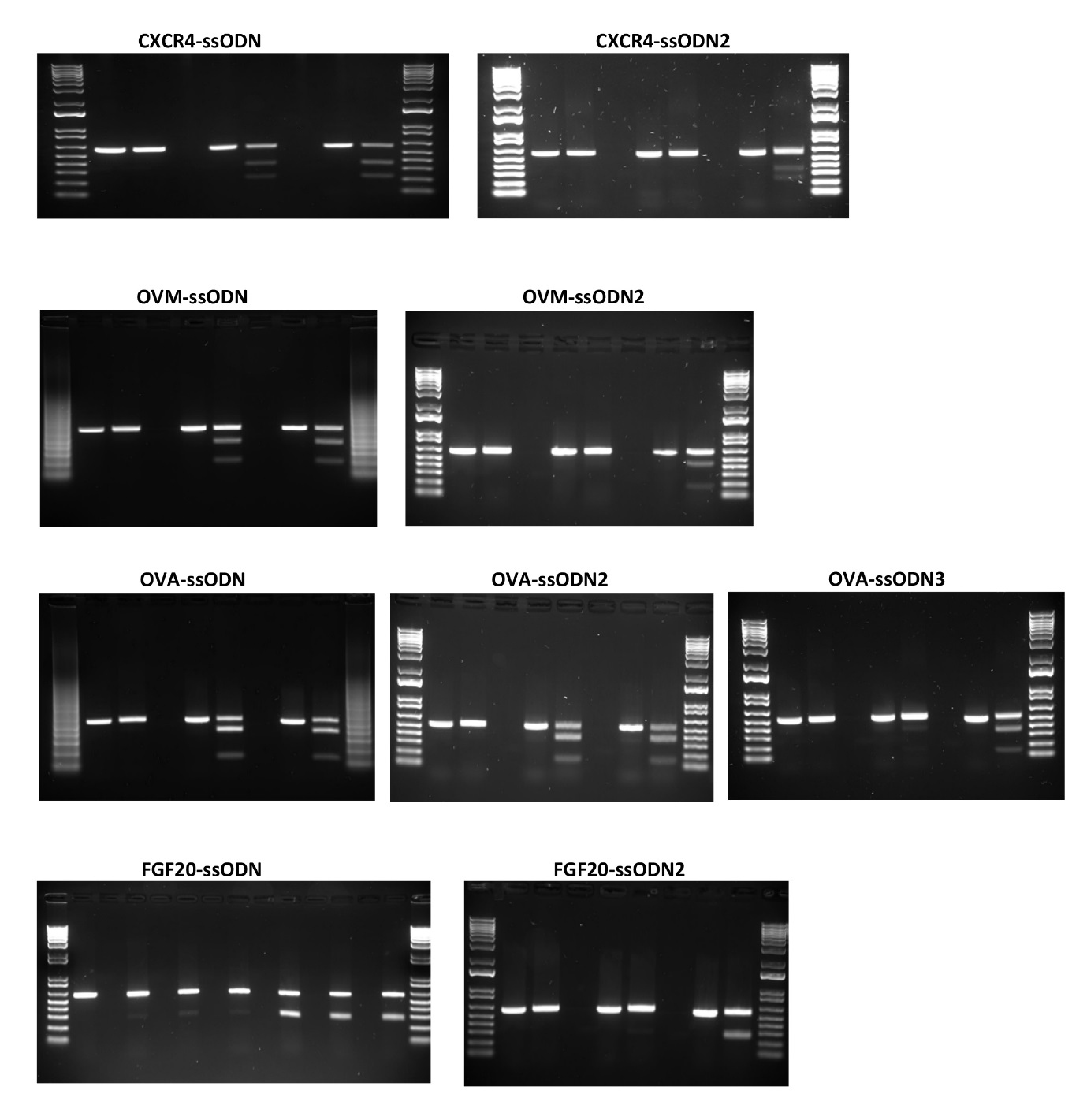


**Figure S9**. Uncropped gel images for Figure 2.


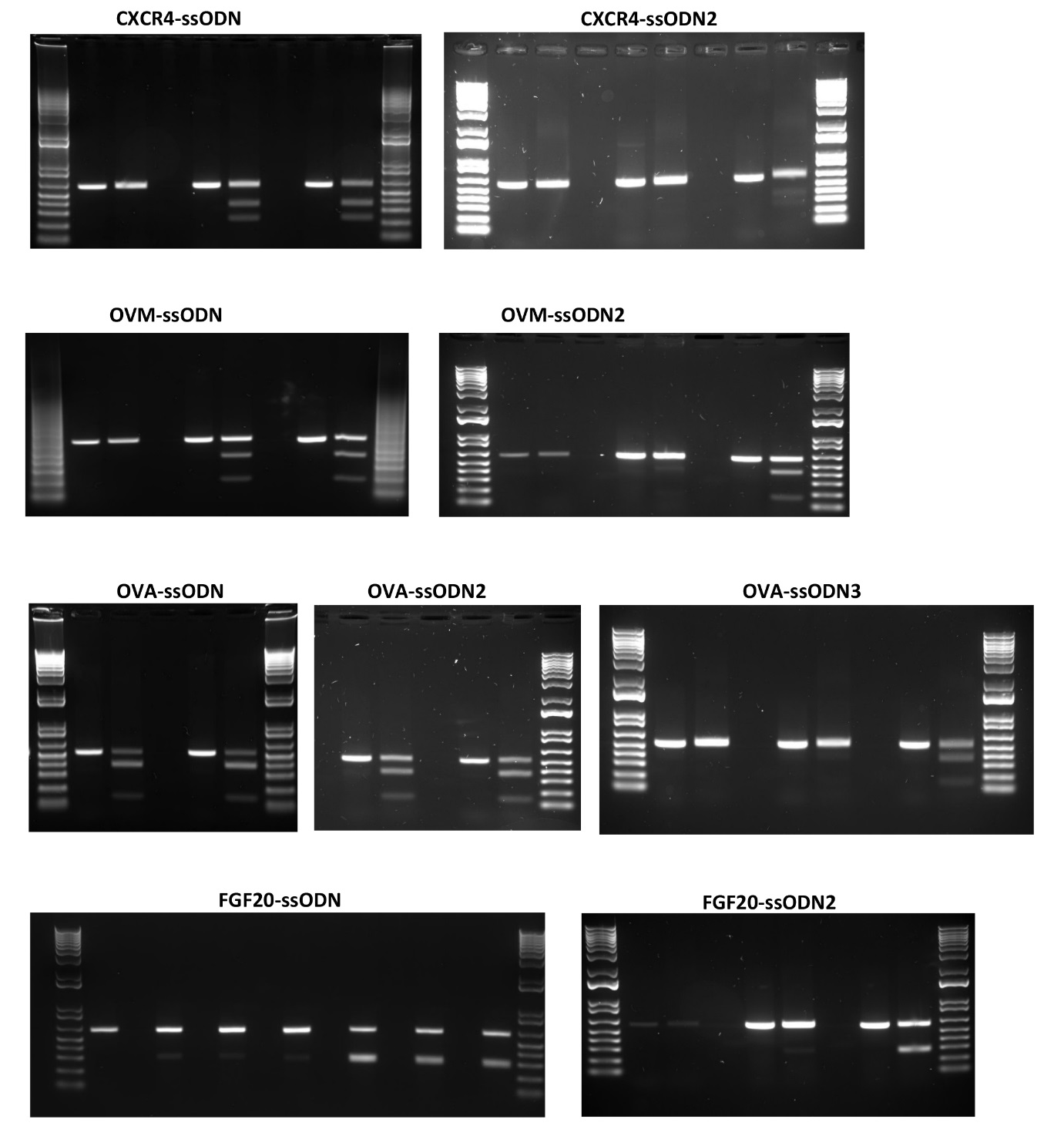


**Figure S10**. Uncropped gel images for Figure S2.

**Table S1**. List of gRNAs

| Name | 5’ 3’ |
| --- | --- |
| CXCR4-gRNA | caccgACAATGGCTCGGAGGAGAT |
| Ovomucoid-gRNA | caccgTTTCCCAACGCTACAGACA |
| Ovalbumin-gRNA | caccgCTCTAGCCATGGTATACCT |
| GFP-gRNA | caccCTCGTGACCACCCTGACCTA |
| FGF20-gRNA1 | caccgTACTCCCAGAGATGGAGCA |
| FGF20-gRNA2 | caccgTGTGAACTTCTGGTGTCTTT |
| FGF20-gRNA3 | caccgCAGGAAATGTGTGAACTTC |

**Table S2**. List of ssODN donor sequences

| **Name** | **5’ 3’** |
| --- | --- |
| CXCR4-ssODN | AAATCAGCGTTTTCATGCTGAAAGCATGGCTCTCCATAGTCTCCATAGTCAGCTGAGCCGAATTCCTCCGAGCCATTAGTCAGCAAATTCAATGAGTATGCCAGAGGACAGCTAAATAAATA |
| CXCR4-ssODN2 | TCAGCGTTTTCATGCTGAAAGCATGGCTCTCCATAGTCTCCATAGTCAGCTGAGCCAATCTCCTCCGAGCCATTGACGTCAAATTCAATGAGTATGCCAGAGGACAGCTAAATAAATAAA |
| OVM-ssODN | ACTCCATCGGTACCACAGATGGGGCGGAGGTCCTTGTTGCAAACCAATACATCTTTGCCTTGAATTCGTCTGTAGCGTTGGGAAACCTACTGCAGTCCACCTGACAAAGAAACACACAGCAACAGGA |
| OVM-ssODN2 | ACTCCATCGGTACCACAGATGGGGCGGAGGTCCTTGTTGCAAACCAATACATCTTTGCCTTCCTTGTCTGTAGCGTTGGGAtcCCTACTGCAGTCCACCTGACAAAGAAACACACAGCAACAGGA |
| OVA-ssODN | GTTTTAATCTTTAACTGTAGGCTCACCTTATTTATCTGTGTCCTGGTGCTGTCTTTTGCAGAATTCTATACCATGGCTAGAGCTGACATGATGGCAATGGGGCAGTAGAAGATGTTCTCATTGGCAT |
| OVA-ssODN2 | GTTTTAATCTTTAACTGTAGGCTCACCTTATTTATCTGTGTCCTGGTGCTGTCTTTTGCACCCAGGTATACCATGGATCCAGCTGACATGATGGCAATGGGGCAGTAGAAGATGTTCTCATTGGCAT |
| OVA-ssODN3 | GTTTTAATCTTTAACTGTAGGCTCACCTTATTTATCTGTGTCCTGGTGCTGTCTTTTGCACCCAGGTATACCATGGCTAGCGCTGACATGATGGCAATGGGGCAGTAGAAGATGTTCTCATTGGCAT |
| FGF20-ssODN | CCAGCACATCTTTATACAGTTCTGGAACTCTTTCAGGATCCACAGGTCTGGGCAGGAAATGTGTGAACTTCTGGTGTCTTTTGGAATTCGCTCCATCTCTGGGAGTACCATCTTTGTTAAGTGCTA |
| FGF20-ssODN2 | TCTTTCAGGATCCACAGGTCTGGGCAGGAAATGTGTGAACTTCTGGTGTCTTTTGGACCTTGCTCCATCTCTGGGAATTCCATCTTTGTTAAGTGCTACGAAGTATCGCCGCCCAGAATCTCCATG |
| BFP-ssODN | GGCATGGCGGACTTGAAGAAGTCGTGCTGCTTCATGTGGTCGGGGTAGCGGCTGAAGCACTGCACCCCGTGGCTCAGGGTGGTCACGAGGGTGGGCCAGGGCACGGGCAGCTTGCCGGTGGTGCAGATGAACTTCAGGGT |
| GFP-ssODN | GGCATGGCGGACTTGAAGAAGTCGTGCTGCTTCATGTGGTCGGGGTAGCGGCTGAAGCACTGCACACCATACGTCAGGGTGGTCACGAGGGTGGGCCAGGGCACGGGCAGCTTGCCGGTGGTGCAGATGAACTTCAGGGT |
| Sca-ssODN | CCAGCACATCTTTATACAGTTCTGGAACTCTTTCAGGATCCACAGGTCTGGGCAGGAAATGTGTGAACTTCTGGTGTCATTTGGATCTTGCTCCATCTCTGGGAGTACCATCTTTGTTAAGTGCTA |
| Silent-ssODN | CCAGCACATCTTTATACAGTTCTGGAACTCTTTCAGGATCCACAGGTCTGGGCAGGAAATGTGTGAACTTCTGGTGTCTTTTGGAGCGTGCTCCATCTCTGGGAGTACCATCTTTGTTAAGTGCTA |
| Silent2-ssODN | CCAGCACATCTTTATACAGTTCTGGAACTCTTTCAGGATCCACAGGTCTGGGCAGGAAATGTGTGAACTTCTGGTGTCGTTTGGATCTTGCTCCATCTCTGGGAGTACCATCTTTGTTAAGTGCTA |

**Table S3**. List of PCR primers

| **Name** | **5’ 3’** |
| --- | --- |
| CXCR4 | Forward: 5’- TGTAGCACGCATCCCATTAGA -3’  Reverse: 5’- AGGTGATGACAAAGAGGAGGT -3’ |
| Ovomucoid | Forward- 5’ – GCTGGTTTATCACATGGGGAC -3’  Reverse- 5’ – CACCTCTCCATCCTTTTGCTC -3’ |
| Ovalbumin | Forward- 5’ – ACCCAAAAGACAACTGAATGCA -3’  Reverse- 5’ – GAGCTATGCAGTTTCCAAGGG -3’ |
| FGF20 | Forward- 5’- TGTCAGGTCTACACACTCCTC-3’  Reverse- 5’- CAAGTTTGAAGGAGGCTGGTC-3’ |
| GFP | Forward- 5’- TAAACGGCCACAAGTTCAGC-3’  Reverse- 5’- GATGTTGTGGCGGATCTTGAA-3’ |
